# Effectiveness of Pandemic and Seasonal Influenza Vaccines in Preventing Laboratory-Confirmed Influenza in Adults: A Clinical Cohort Study during Epidemic Seasons 2009–2010 and 2010–2011 in Finland

**DOI:** 10.1371/journal.pone.0108538

**Published:** 2014-09-29

**Authors:** Ritva K. Syrjänen, Jukka Jokinen, Thedi Ziegler, Jonas Sundman, Mika Lahdenkari, Ilkka Julkunen, Terhi M. Kilpi

**Affiliations:** 1 Department of Vaccination and Immune Protection, National Institute for Health and Welfare, Tampere, Finland; 2 Department of Vaccination and Immune Protection, National Institute for Health and Welfare, Helsinki, Finland; University of Pittsburgh, United States of America

## Abstract

**Background:**

One dose of pandemic influenza vaccine Pandemrix (GlaxoSmithKline) was offered to the entire population of Finland in 2009–10. We conducted a prospective clinical cohort study to determine the vaccine effectiveness in preventing febrile laboratory-confirmed influenza infection during the influenza season 2009–10 and continued the study in 2010–11.

**Methods:**

In total, 3,518 community dwelling adults aged 18–75 years living in Tampere city were enrolled. The participants were not assigned to any vaccination regimen, but they could participate in the study regardless of their vaccination status or intention to be vaccinated with the pandemic or seasonal influenza vaccine. They were asked to report if they received Pandemrix in 2009–10 and/or trivalent influenza vaccine in 2010–11. Vaccinations were verified from medical records. The participants were instructed to report all acute symptoms of respiratory tract infection with fever (at least 38°C) and pneumonias to the study staff. Nasal and oral swabs were obtained within 5–7 days after symptom onset and influenza-specific RNA was identified by reverse transcription polymerase chain reaction.

**Results:**

In 2009–10, the estimated vaccine effectiveness was 81% (95%CI 30–97). However, the vaccine effectiveness could not be estimated reliably, because only persons in prioritized groups were vaccinated before/during the first pandemic wave and many participants were enrolled when they already had the symptoms of A(H1N1)pdm09 influenza infection. In 2010–11, 2,276 participants continued the follow-up. The vaccine effectiveness, adjusted for potential confounding factors was 81% (95%CI 41–96) for Pandemrix only and 88% (95%CI 63–97) for either Pandemrix or trivalent influenza vaccine 2010–11 or both, respectively.

**Conclusion:**

Vaccination with an AS03-adjuvanted pandemic vaccine in 2009–10 was still effective in preventing A(H1N1)pdm09 influenza during the following epidemic season in 2010–11.

**Trial Registration:**

ClinicalTrials.gov NCT01024725. NCT01206114.

## Introduction

The disease burden of influenza virus infections is huge. The antigenic variation of the virus and waning herd immunity lead to yearly influenza epidemics in most parts of the world. If a new reassortant influenza virus has the ability for human-to-human transmission the virus may spread very rapidly and effectively throughout the world, since most individuals lack protective immunity against the new virus. This happened in the spring of 2009, when a swine origin A(H1N1)pdm09 influenza virus caused the first pandemic of the 21^st^ century. Although the overall case fatality of the A(H1N1)pdm09 influenza was lower than initially observed in Mexico [1]–[3], the epidemic caused a considerable disease burden. The age-standardized cumulative incidence during the first year was approximately 24% when estimated using serological data from 19 countries/regions and globally there were more than 200,000 respiratory deaths with an additional 83,300 cardiovascular deaths associated with A(H1N1)pdm09 influenza [1], [4]. A special feature of the disease as compared with the yearly seasonal influenza epidemics was that it affected younger age groups, whereas the elderly had some cross-reactive immunity resulting from infections with the Spanish flu and its descendant viruses [1], [4]–[6].

**Figure 1:**
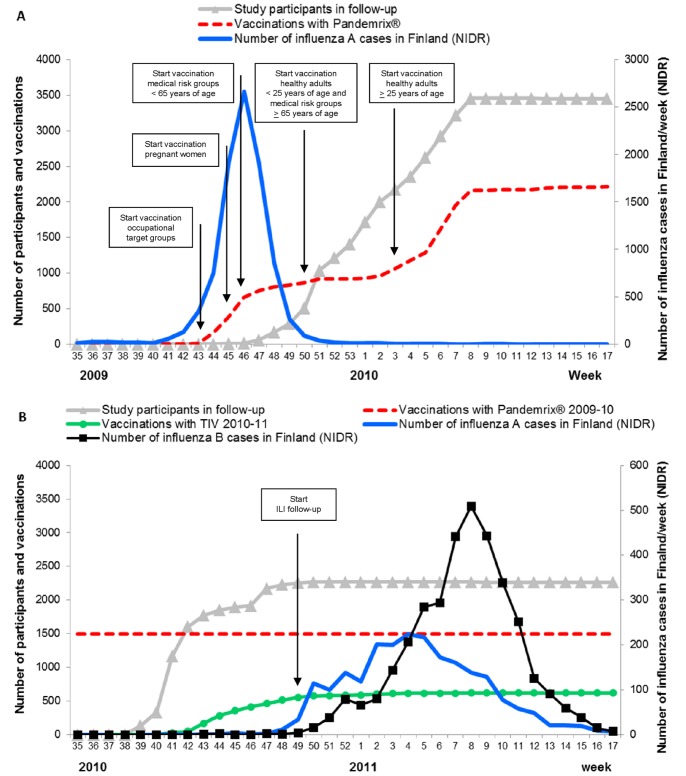
Schematic representation of timing of epidemics, study follow-up and vaccinations. Time course of influenza epidemics in Finland according to the National Infectious Disease Registry (NIDR) and the follow-up of study participants during the epidemic seasons 2009–10 (panel A) and 2010–11 (panel B). Note differences in scales. The main circulating influenza A virus during both epidemic seasons was A(H1N1)pdm09; in 2009–10 only sporadic H3N2 and few influenza B virus findings were observed and during 2010–11>95% of the influenza A strains were A(H1N1)pdm09. The figure shows the vaccination times of the study participants with Pandemrix and seasonal trivalent influenza vaccine (TIV) in 2010–11. The national prioritization order for pandemic vaccination (here shown for adults) in 2009–10 was determined on medical grounds.

The first epidemic of the pandemic A(H1N1)pdm09 influenza in Finland was seen in the fall of 2009 (Figure 1A). It started in October and was practically over by mid-December 2009. During the first epidemic season 7,669 laboratory-confirmed A(H1N1)pdm09 cases were identified in the whole country [7]. Only sporadic H3N2 and few influenza B virus infections were seen [8]. Influenza-associated hospitalization rates were higher during the pandemic as compared with pre-pandemic influenza seasons for persons up to 64 years [9]. The second A(H1N1)pdm09 influenza epidemic occurred between late November 2010 and March 2011 (Figure 1B). The number of laboratory-confirmed influenza cases identified in Finland during this season was 5,767, of which 61% were identified as influenza B and 39% as influenza A. Nearly all (>95%) influenza A viruses typed in the Virology Unit at the National Institute for Health and Welfare (THL) were of the A(H1N1)pdm09 type and the rest were of the seasonal A(H3N2) type [10].

Monovalent pandemic A(H1N1)pdm09 vaccines were used for the prevention of A(H1N1)pdm09 influenza as soon as they became available in late 2009. The estimated effectiveness of monovalent pandemic vaccines against laboratory-confirmed A(H1N1)pdm09 influenza during the epidemic season 2009–10 has varied between 56% and 97% in studies conducted in Europe, US and Canada [11]–[21]. The estimates for the residual effectiveness of monovalent pandemic vaccines administered in 2009–10 against A(H1N1)pdm09 influenza during the following season 2010–11 vary even more [22]–[29].

From October 2009 to May 2010, all residents of Finland were offered one dose of a monovalent AS03-adjuvanted pandemic A(H1N1)pdm09 vaccine (Pandemrix, GlaxoSmithKline Biologicals) as part of the national vaccination campaign. The order for vaccination was determined on medical grounds by the Finnish government, prioritizing the health and social care workers working with influenza patients or patients exposed and vulnerable to infections, pregnant women, persons less than 65 years of age with an underlying medical condition and healthy children and young adults (Table S1, 30). Vaccination was voluntary and free of charge. The mean coverage in all age groups was 52%, being highest in children less than 15 years of age (74–82%) and lowest in young adults aged 20–29 years (32%) [30]. For the 2010–11 influenza season, the trivalent influenza vaccine (TIV) was offered to young children, pregnant women, medical risk groups, and healthcare professionals.

We conducted a prospective, observational clinical cohort study from November 2009 to April 2010 and continued it from December 2010 to April 2011 to determine the effectiveness of vaccination in preventing febrile laboratory-confirmed infection caused by the pandemic A(H1N1)pdm09 influenza virus among community-dwelling adults in Finland. During the epidemic season 2010–11, we also assessed the effectiveness of TIV against influenza B.

## Materials and Methods

### Study population and enrolment

All residents of Tampere city were eligible to participate in the study, if they were 18–75 years of age, community-dwelling, with full legal competence and able to communicate fluently in Finnish or Swedish. Invitation letters were sent home to addresses retrieved from the Population Register Centre, and distributed to pregnant women at maternity clinics and to healthcare professionals at work. In addition, announcements were published in local newspapers. Persons who volunteered to participate in the study were invited to special clinics established for the study to a baseline visit, during which written informed consent was obtained and background information was gathered with structured questionnaires.

In September 2010, all subjects having complied with the follow-up in the study 2009–10 and still living in Tampere were invited to participate in the second phase of the study through letters that were sent to their home addresses. All participants were asked to give written consent and return a questionnaire with updated background data.

### Vaccinations

No vaccines were administered in the study and the participants were not assigned to any vaccination regimen, but they could participate in the study regardless of their vaccination status or intention to be vaccinated with the pandemic or seasonal influenza vaccine.

The only pandemic vaccine available in Finland (Pandemrix) contained inactivated, split influenza virus propagated in eggs and an oil-in-water adjuvant AS03 [31]. Local healthcare centers were responsible for the vaccinations and they were obliged to record all pandemic vaccination events in electronic patient record systems. These events are individually identifiable via a unique personal identity code, assigned to all permanent residents in Finland. The subjects could participate in the study regardless of their vaccination status or intention to be vaccinated with the pandemic or seasonal influenza vaccine.

Pandemrix was not recommended for the epidemic season 2010–11. Instead, the seasonal unadjuvanted TIV (either Fluarix or Vaxigrip) including A/California/07/2009(H1N1)-derived virus antigen was offered free of charge and administered in local healthcare centers as part of the national vaccination program for adults belonging to medical risk groups (Table S1), for pregnant women, for persons at least 65 years of age and frontline health and social care workers. In addition, several TIV products were available in private retail pharmacies and medical centers. During the epidemic season 2010–11, the four possible A(H1N1)pdm09 influenza vaccination regimens were: 1) only one dose of Pandemrix during the vaccination campaign in 2009–10; 2) only one dose of any of the TIVs for the season 2010–11; 3) one dose of Pandemrix in 2009–10 and one dose of TIV for the season 2010–11; 4) no vaccination with any of A(H1N1)pdm09 influenza virus-containing vaccines.

Data on the vaccination status and date of vaccination were derived from the medical records of the healthcare center of Tampere city. In addition, the participants were asked in the beginning of both phases of the study, whether they had received Pandemrix. At the start of the second phase the participants were asked whether they had already received TIV for the season 2010–11. In addition, they were asked to return a vaccination card signed by the vaccinator, if they took TIV later. The participants were also asked monthly by text messages, phone or e-mail, whether they had taken TIV. All self-reports on receipt of Pandemrix and/or TIV 2010–11 were verified from the medical records of the vaccinators whenever possible. After vaccination, the participant was considered unvaccinated, until 14 days had elapsed since vaccination and thereafter, vaccinated.

### Case definitions

Influenza-like illness (ILI) was defined as a sudden onset of the following self-reported clinical signs and symptoms: measured fever (≥38°C) and at least one sign or symptom of acute respiratory infection. In addition, pneumonia diagnosed by a physician was also regarded as an ILI. The definition was adapted from the clinical criteria for the novel influenza virus A(H1N1)pdm09 infection as outlined in the European Commission Decision 2009/363/EC [32].

Nasal and/or throat swab specimens were collected within 5 days after the onset of symptoms from patients suffering from an ILI episode. During the second phase of the study, the sampling window after onset was extended for logistical reasons to 7 days. This sampling window, commonly used in influenza surveys was chosen after analysis of the performance of the used reverse transcription polymerase chain reaction (RT-PCR) method in the Virology Unit at THL (author’s unpublished data). The patients were considered to have a laboratory-confirmed influenza infection if the samples were positive for influenza A or B virus RNA by RT-PCR method [33]. If the samples were positive for influenza A RNA, but negative in subtype A(H1N1)pdm09-specific RT-PCR assay the patient was considered to have a seasonal H3N2 type infection.

### The follow-up and clinical samples

During the first epidemic season, the recruitment and follow-up started on the 3^rd^ of November 2009, shortly before the epidemic peak in the study area, and lasted up to 30^th^ April 2010. During the second season, the follow-up started at the onset of the second A(H1N1)pdm09 influenza epidemic on 8^th^ December 2010 when a signal in the National Infectious Disease Registry (NIDR) had been identified as defined in the study protocol, and lasted up to 30^th^ April 2011.

The participants were instructed to contact study staff immediately if they had symptoms of ILI as defined above. In addition, the participants were asked to fill in a diary to define the onset time of symptoms and to characterize the clinical features of the disease. To enhance the compliance, the participants were contacted weekly by text messages, phone or e-mail and asked to reply to a question of possible symptoms of ILI.

Whenever a study subject reported suffering from ILI defined above, an acute phase visit was arranged either at home or at a special study clinic, if it was possible to perform within 5 days (in 2010–11, 7 days) after the onset of symptoms. During these visits, samples were collected by a qualified study nurse or physician from both nostrils with a nylon-tipped flocked swab (Copan) and from the throat with a similar, fresh swab. Both swabs were placed into a tube containing transport medium (Copan, Universal Transport Medium UTM-RT). In 2009–10, samples were frozen (≤−70°C) until analyzed and in 2010–11, they were sent to the laboratory twice a week and analyzed immediately. The vaccination status of the subjects was known to themselves and to the study personnel obtaining the samples but not to the personnel performing the microbiological analyses. Information on potential missed cases was collected from NIDR, to which all Finnish clinical microbiology laboratories are obliged to report findings of positive influenza cases.

### Statistical methods

The study was powered to assess the effectiveness of Pandemrix against A(H1N1)pdm09 influenza infection. For sample size considerations, vaccine effectiveness of 70% was assumed. For the first (2009–2010) and second (2010–2011) phases of the study, the attack rates of A(H1N1)pdm09 influenza in the unvaccinated were assumed, respectively, 20% and 5%, and the vaccine coverages as 90% and 50%. The study was calculated to achieve a power of 95% with a sample size of 4000 in 2009–10 and 3000 in 2010–11. High power was preferred because of potential confounding factors and uncertainty in the assumptions. Statistical software R (version 3.02) [34] was used for statistical analysis.

The incidence of laboratory-confirmed A(H1N1)pdm09 pandemic influenza was calculated as the number of cases among vaccinated and unvaccinated groups divided by the person-time at risk. The relative risk between vaccinated and unvaccinated individuals was estimated using Poisson regression, where the person-times in respective groups were used as weights in the model. Vaccine effectiveness (VE) was calculated as 1-relative risk. Profile likelihood method was used for estimating the 95% confidence interval (95%CI) for the vaccine effectiveness. Age-group (18–49 years, 50–75 years), gender, underlying medical conditions (Table S1) and pregnancy were included as covariates when calculating the estimates for adjusted vaccine effectiveness, based on information collected in the background questionnaires. Vaccine attributable reduction was calculated as a cumulative incidence of A(H1N1)pdm09 cases among the vaccinated individuals minus the expected cumulative incidence without vaccination during the same follow-up time.

### Ethics statement

The studies were undertaken in compliance with applicable regulatory requirements and Declaration of Helsinki. The study protocols were approved by the ethics committee of the Pirkanmaa Hospital District. Written informed consent was obtained from all study participants at enrolment in the first study phase 2009–10 and all participants who volunteered to participate in the second phase 2010–11 were asked to send written consent by mail.

The studies were registered as observational studies in ClinicalTrials.gov (NCT01024725, NCT01206114). The protocols for the studies and supporting TREND checklist are available as supporting information; see Protocol S1 (Study protocol 2009–10), Protocol S2 (Study protocol 2010–11) and Checklist S1 (TREND checklist). In addition to the objectives determined in the protocols, the effectiveness of TIV against influenza B was estimated, because influenza B was the most frequently circulating influenza virus during the epidemic season 2010–11.

## Results

### Results of the 2009–2010 season

**Figure 2:**
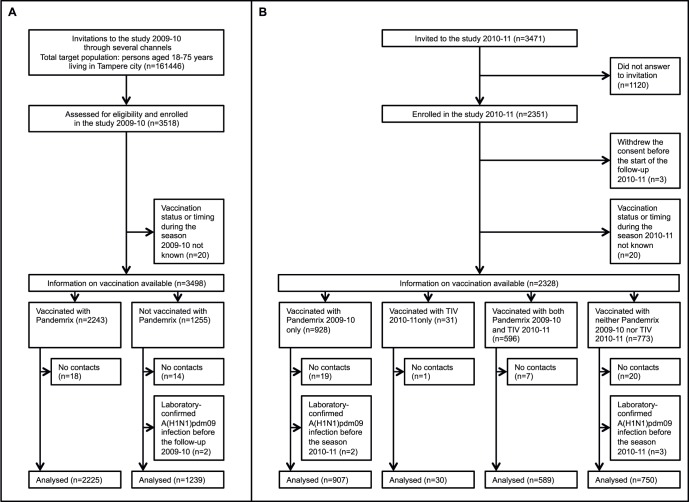
Flow chart of data selection. The figure illustrates the exclusion and inclusion of participants for the analysis of the effectiveness of pandemic and seasonal influenza vaccines in preventing laboratory-confirmed influenza in Finnish adults: a clinical cohort study. A, the study during the epidemic season 2009–10. Invitation letters were sent home and distributed to pregnant women at maternity clinics and to healthcare professionals at their work place. In addition, announcements were published in newspapers. Of the 3,464 participants followed and vaccination status known during 2009–10, 2,731 were enrolled after the first epidemic peak in 2009. Of the 32 subjects without contacts 2009–2010, 22 had failed to receive text messages or phone calls. They were contacted after the follow-up and invited to the follow-up 2010–11. B, the study during the epidemic season 2010–11.

Between 3^rd^ of November 2009 and 26^th^ of February 2010, 3,518 participants were enrolled in the study. Table S2 shows the background data of these participants. Of them, 32 could not be contacted after the baseline visit, two had a laboratory-confirmed A(H1N1)pdm09 influenza infection before enrolment and for an additional 20, the timing of vaccination with Pandemrix remained unknown. All these 54 participants were excluded from the analysis (Figure 2A). None of the excluded participants was known to have had influenza during the study period. Of the 3,464 participants included in the analysis, 3,374 responded to 96% of the weekly sent text messages and 90 participants were followed by phone. Eleven participants discontinued the follow-up before the end of the study.

Altogether, 2,225 (64%) of the 3,464 participants were vaccinated with Pandemrix (first vaccination 19^th^ October 2009, last 26^th^ May 2010, Figure 1A) according to vaccinations recorded in the electronic patient record systems (N = 2175), and additional information on received vaccination obtained credibly from the subjects through the study questionnaires (N = 50).

Both nasal and oropharyngeal swab samples were obtained during all 112 ILI-related study visits. A(H1N1)pdm09 influenza virus was identified in 13 of the samples. Of the 13 cases, two occurred in vaccinated individuals and 11 occurred in unvaccinated persons, during 6,078 and 6,253 months of person-time at risk, respectively. The estimated VE was thus 81% (95%CI 30–97). No missed cases of influenza were discovered in the NIDR among the study participants during the study period.

Of the study participants, 733 were enrolled before the end of the epidemic peak, out of whom 227 (31%) were vaccinated before mid-December 2009. Of these 733, 427 (58%) belonged to at least one priority target group for vaccination. All 13 A(H1N1)pdm09 influenza cases identified in the study occurred before mid-December 2009, and 11 of the patients belonged to one or more of the priority target groups for vaccination. Moreover, 7 of the 13 A(H1N1)pdm09 patients volunteered to participate at the time he/she already had symptoms of the disease.

### Results of the 2010–2011 season

Altogether 3,471 participants who had complied with the follow-up in the 2009–10 study and were still living in Tampere were invited to the follow-up in 2010–11. Of them, 2,351 volunteered to participate between 30^th^ of September 2010 and 14^th^ of February 2011. Figure 2B provides the reasons for exclusion of 75 subjects. These subjects were similar to those included as regards the age, gender, pregnancy, medical risk group and vaccination status (if known). None of them had laboratory-confirmed influenza during the season 2010–11.

Of the 2,276 participants included in the final analysis, 2,214 replied to 91% of the text messages sent monthly to monitor influenza vaccinations and to 96% of those sent weekly to get information on potential current symptoms of ILI. The remaining 62 participants were followed by regular phone calls or e-mails.

The 2,276 participants were followed for altogether 10,640 person months (Figure 1B). Table 1 shows the background data of these participants. Of them, 907 (40%) were vaccinated with Pandemrix only, 30 (1%) were vaccinated with TIV 2010–11 only, 589 (26%) were vaccinated with both Pandemrix and TIV 2010–11, and 750 (33%) were not vaccinated with either Pandemrix or TIV 2010–11. Of vaccinations with Pandemrix and TIV 2010–11, 99% and 96% were verified from medical records, respectively. The forty participants whose credible self-reported vaccination could not be verified were considered as vaccinated in the analyses. None of these 40 individuals had influenza during the follow-up.

**Table 1:** Background data of participants included in the analysis during the epidemic season 2010–11, according to vaccination with Pandemrix in 2009–10 and/or trivalent influenza vaccine (TIV) in 2010–11.

		Vaccination status, No (%)				
		All	Pandemrix in 2009–10 only	TIV in 2010–11 only	Both Pandemrix and TIV	Neither
All		2276	907	30	589	750
Age, years^1^	18–24	85 (4)	42 (5)	2 (7)	6 (1)	35 (5)
	25–49	793 (35)	321 (35)	9 (30)	140 (24)	323 (43)
	50–64	1158 (51)	484 (53)	14 (47)	321 (54)	339 (45)
	≥65	240 (11)	60 (7)	5 (17)	122 (21)	53 (7)
Females		1473 (65)	593 (65)	21 (70)	377 (64)	482 (64)
Pregnant^2^		23 (2)	8 (1)	0	8 (2)	7 (1)
Medical target group for vaccination^3^		417 (18)	110 (12)	6 (20)	243 (41)	58 (8)

The vaccination status was determined with information given by the study participants during the two study phases 2009–11 and with information from the medical records of the health center of Tampere city or the vaccinators.

1 Age at start of the follow-up.

2 Pregnant at enrolment, No (% of women). Information on the pregnancy was not available for 2 women vaccinated with both Pandemrix and TIV and for one unvaccinated woman.

3 Individuals with at least one of the following underlying medical conditions: a heart or lung disease requiring regular medication, a metabolic disease, chronic liver failure or chronic kidney disease, an immune system disease, a condition whose treatment reduces the immune response, or a chronic neurological or neuromuscular disease.

Both nasal and oropharyngeal samples were obtained during all of the 172 ILI-related visits, although during three visits, only one nostril was swabbed. Influenza virus RNA was found in 78 (45%) of the samples. The detected virus was A(H1N1)pdm09 in 16 cases (9% of all samples). Seasonal influenza A(H3N2) virus was found 9 times (5%) and influenza B virus 53 times (31%). No additional influenza cases were discovered in the NIDR among the study participants during the study period.

The incidence of A(H1N1)pdm09 influenza among the unvaccinated was 17.3 cases per 1,000 persons during the 2010–11 epidemic season. Of the 16 participants with A(H1N1)pdm09 influenza, three had been vaccinated only with Pandemrix during the previous season 2009–10, and 13 had not been vaccinated with any of the A(H1N1)pdm09 influenza vaccines. In all 16 cases vaccination was verified from medical records.

When adjusted for possible confounding factors, the effectiveness of vaccination with Pandemrix only given in 2009 or early 2010 in preventing A(H1N1)pdm09 influenza during the epidemic season 2010–11 was 81% (95%CI 41–96) (Table 2). The effectiveness was 88% (95%CI 63–97) for either Pandemrix 2009–10 or TIV 2010–11 or both, and 100% (95%CI 79–100) for both Pandemrix and TIV. Unadjusted effectiveness estimates were essentially the same. The reduction in the number of A(H1N1)pdm09 influenza infections attributable to Pandemrix only was 1,454 (95%CI 490–2,419) per 100,000 vaccinated during the second epidemic season in 2010–11.

**Table 2:** The effectiveness of vaccination with Pandemrix in 2009–10 and/or trivalent influenza vaccine (TIV) in 2010–11 in preventing A(H1N1)pdm09 influenza infection during the epidemic season 2010–11.

	Persons	Person monthsat risk	Cases of A(H1N1)pdm09 influenza	Vaccine effectiveness(95%CI)	Adjusted vaccineeffectiveness^1^(95%CI)
Pandemrix in 2009–10 only	907	4333	3	81.5% (42.5–95.8)	81.2% (41.4–95.7)
TIV in 2010–11 only	30	129	0	100.0% (−328.9–100.0)	100.0% (−329.9–100.0)
Both Pandemrix and TIV	589	2652	0	100.0% (79.1–100.0)	100.0% (78.6–100.0)
Either Pandemrix orTIV or both	1526	7114	3	88.7% (65.0–97.4)	88.4% (62.8–97.4)
Neither	750	3479	13	reference	reference

Profile likelihood method was used for estimating the 95% confidence intervals (95%CI) for the vaccine effectiveness.

1 Adjusted for the age group (18–49, 50–75 years), gender, underlying medical condition and pregnancy.

Of the 53 cases of influenza B, 8 were observed during 2,766 person months at risk in individuals vaccinated with TIV 2010–11 and 45 were observed during 7,769 person months in those not vaccinated with TIV 2010–11. The adjusted and unadjusted VE estimates of the seasonal TIV in preventing influenza B virus infections were 55% (95%CI 5–81) and 50% (95%CI 0–78), respectively. Of the 9 influenza A(H3N2) cases, 4 occurred in TIV-vaccinated individuals. Apparently, TIV gave no or poor protection against non-A(H1N1)pdm09 infections, but the total number of cases was too low for an appropriate analysis.

## Discussion

We followed a large cohort of adults in a prospective clinical cohort study during the first and second epidemic seasons of A(H1N1)pdm09 influenza in Finland and found that the effectiveness of one dose of an AS03-adjuvanted monovalent vaccine (Pandemrix) administered between October 2009 and May 2010, remained high, being 81% (95%CI 41–96) against laboratory-confirmed A(H1N1)pdm09 influenza virus infection during the second, 2010–11 epidemic season. Vaccination with both Pandemrix in 2009–10 and TIV in 2010–11 provided excellent protection (VE 100%). During the first epidemic season 2009–10, the VE estimate for Pandemrix was also 81%. However, the VE estimate for the season 2009–10 should be considered with caution, since only persons belonging to the high priority vaccination groups were vaccinated before the pandemic peak was over (Figure 1A) and many participants were enrolled when they already had the symptoms of A(H1N1)pdm09 influenza infection.

The residual effectiveness of the AS03-adjuvanted monovalent pandemic vaccine administered during the epidemic season 2009–10 without any additional vaccination with TIV in 2010–11 against A(H1N1)pdm09 influenza in 2010–11 in adults was higher in the current study than in most other published studies. Also in Canada, where an AS03-adjuvanted vaccine (Arepanrix), similar to Pandemrix constituted about 95% of the pandemic vaccine distributed in 2009 [19], a relatively high residual VE of 69% (95%CI, 38–85) overall and 76% (95%CI, 42–90) among young adults was found in 2010–11 [23]. Instead, no prevailing effectiveness was found for vaccination with either unadjuvanted inactivated (VE -1, 95%CI -146–59) or live attenuated monovalent (VE -13, 95%CI -265–65) pandemic vaccines in 2009–10 against A(H1N1)pdm09 influenza during the season 2010–11 in the US [22]. In other studies, intermediate 28–66% residual VE estimates for pandemic vaccine 2009–10 in all age groups was found in areas that used mainly AS03-adjuvanted vaccines [24], [25], [27]. VE of 56% was seen in Australian adults for an unadjuvanted, inactivated, split-virus vaccine about 7 months after pandemic vaccination [26]. None of these estimates, however, reached statistical significance.

It is noteworthy that also during the first A(H1N1)pdm09 influenza epidemic season in 2009–10, high effectiveness has been demonstrated for AS03-adjuvanted vaccines in many studies, which is in good accordance with the high immunogenicity elicited by these vaccines [31], [35]. In British Columbia, Alberta, Ontario, and Quebec in Canada, the adjusted VE in all age groups was 93% (95%CI 69–98) in a test-negative case-control setting [19]. The high effectiveness of the vaccine used in Canada was confirmed by another group in a population based case–control study in Manitoba (VE 86%, 95%CI 75–93) [36]. In Sweden, the estimated (weekly) VE was 69–89% during the maximum influenza activity in adults 30–64 years of age, and even higher in children [17]. In Norway, the weekly VE during the epidemic ranged from 77% to 96% in a population-based retrospective cohort study combining the disease surveillance and vaccination registers [37]. High VE (>90%) has also been shown for mainly AS03-adjuvanted pandemic vaccine in Germany among 14–59-year-old individuals and among Portuguese healthcare workers [15], [18]. Intermediate VE estimates (67–74%) in 2009–10 were reported in persons more than 14–15 years of age from UK and Germany, countries which used mainly AS03-adjuvanted vaccines [14], [16]. These estimates are comparable with the pooled adjusted VE of 66–73% in persons aged 15–64 years in 7 European countries using different types of vaccines [11]. In the US, VE was 89% (95%CI 15–99) for three unadjuvanted inactivated vaccines in persons aged 10–49 years, but no significant effectiveness was seen in older adults, and an unadjuvanted live attenuated vaccine did not show any significant effectiveness even in the age group of 10–49-year-old individuals [12]. Finland, Canada, Sweden and Norway had a universal vaccination policy with high coverage [30], [38], [39] whereas in many countries mainly risk groups were targeted, which may explain lower VE estimates [11], [17], [29], [36], [40].

In the current study, no A(H1N1)pdm09 influenza cases were seen during the season 2010–11 among participants vaccinated with both Pandemrix in 2009–10 and TIV in 2010–11. However, the 95% confidence intervals for the VE for different combinations of Pandemrix and TIV overlapped. Several other studies show that vaccination with both monovalent pandemic vaccine 2009–2010 and TIV 2010–11 yield higher point estimates for protection than either of the vaccines alone [23]–[24], [27], although the confidence intervals overlap and different results have also been found [22], [28]. The VE of the TIV 2010–11 only against A(H1N1)pdm09 influenza could not be reliably assessed, since this group included only 30 individuals.

The observed effectiveness of the TIV 2010–11 against influenza B virus has varied from 23 to 69%, with considerable variation between age groups and study sites and with wide confidence intervals, often including zero [23]–[25], [27], [28]. One reason for this variation may be the different local distribution of viruses in the Victoria and Yamagata lineages. In a UK study, the VE against viruses in the Victoria lineage was 78% (95%CI 51–91) when the vaccine strain was from the same lineage whereas no significant effectiveness was observed against those in the Yamagata lineage [24]. In the current study, the adjusted VE of TIV 2010–11 against influenza B was 55% (95%CI 5–81). The influenza B strains were not further characterized, but viruses from both lineages were circulating in Finland during that season [8].

Differences in care-seeking habits in the target populations, in the clinical outcomes, in the registration routines and other methodological aspects may affect the VE in observational studies [41]. The current study was a prospective clinical cohort study, in which participants were well instructed and committed to report all predefined ILI symptoms timely to a qualified study staff, and the occurrence of symptoms were actively monitored by frequent contacts. Therefore, we consider that the methodology in our study was optimal for provision of a reliable estimate for the vaccine effectiveness against febrile A(H1N1)pdm09 influenza virus infection. A shortcoming was, that we did not know, whether the participants had suffered from A(H1N1)pdm09 influenza infection before the follow-up, since no serological data was available. However, it seems unlikely that this could have led to significant overestimation of the vaccine effectiveness. The participants with high probability to contract influenza, i.e. 18–24-year-old individuals and health or social care workers with contacts with influenza patients (part of the occupational target group) ranked high in prioritization order for vaccination with Pandemrix, and were thus likely to have higher probability of having a history of both A(H1N1)pdm09 influenza infection and vaccination during the epidemic in 2009–10 than the other participants. However, the potential additive immunological effect of natural infection and vaccination was likely to be offset by their higher susceptibility or exposure to infection. Furthermore, during the epidemic season 2010–11, only a small minority of participants belonged to these target groups (Table S2) and two thirds of all participants were vaccinated against A(H1N1)pdm09 influenza. Individuals who had already had the infection and were thereby not any more susceptible were hardly more likely to take the vaccine than those who had not yet had symptoms of influenza during the epidemic.

According to the current study, vaccination with Pandemrix 2009–10 was still very effective during the 2010–11 epidemic season in preventing febrile A(H1N1)2009 influenza infection. From the estimate of vaccine attributable reduction it can be extrapolated that vaccination with Pandemrix prevented approximately 40,000 cases of A(H1N1)pdm09 infection during the epidemic season 2010–11 among the total Finnish population of 5.3 million. It is likely that the high vaccination coverage in 2009–10 still gave relatively good population level protection against the A(H1N1)pdm09 virus during the second epidemic season in 2010–11 leading to clearly lower epidemic activity (Figure 1A vs 1B). This was seen especially in children less than 15 years of age, who had the highest attack rate in 2009–10, the highest coverage of vaccination with Pandemrix soon after the epidemic peak 2009 and the most significant decline in the attack rate between 2009–10 and 2010–11. Teenagers also had a high attack rate in 2009–10 but lower vaccination coverage and thus a smaller decline in the attack rate between 2009–10 and 2010–11 was seen [10], [42]. This was not the case in UK, Denmark, Greece and Taiwan, where the disease burden with severe cases was even higher in 2010–11 as compared to the first A(H1N1)pdm09 influenza epidemic season [43]–[46]. We conclude that efficient vaccination strategies and protocols for measuring their effectiveness are needed also for future pandemics.

## Supporting Information

Table S1 **The prioritization order of pandemic vaccination in Finland 2009–10.**
(DOC)

Table S2 **Background data of the study participants.**
(DOCX)

Protocol S1 **Study protocol 2009–10.**
(PDF)

Protocol S2 **Study protocol 2010–11.**
(PDF)

Checklist S1 **TREND checklist.**
(PDF)
