# Supplementary material for: Effectiveness of Pandemic and Seasonal Influenza Vaccines in Preventing Laboratory-Confirmed Influenza in Adults: A Clinical Cohort Study during Epidemic Seasons 2009–2010 and 2010–2011 in Finland
Source: PLoS One. 2014 Sep 29;9(9):e108538. doi: 10.1371/journal.pone.0108538 (PMC4180439; doi:10.1371/journal.pone.0108538)
Supplement: Table S1 — The prioritization order of pandemic vaccination in Finland 2009–10. (DOC) [file pone.0108538.s001.doc]

**Table S1**

**The prioritization order of vaccination against pandemic influenza A(H1N1)pdm09** in Finland 2009-10

| 1. Frontline health and social care workers treating and caring for infected patients or patients exposed to infections^1^ |
| --- |
| 2. Pregnant women |
| 3. Persons aged from 6 months to 64 years who due to an underlying medical condition belong to a risk group^2^ |
| 4. Healthy children aged from 6 to 35 months |
| 5. Healthy children and young individuals aged from 3 to 24 years as well as military conscripts |
| 6. Persons aged 65 and over who due to an underlying medical condition belong to a risk group^2^ |
| 7. Healthy individuals aged 25 and over |

^1^Including ambulance staff and pharmacy staff working in customer service. Patients exposed to infection included those with high risk to develop severe infection or complications.

^2^The medical conditions comprised a heart or lung disease requiring regular medication, a metabolic disease, chronic liver failure or chronic kidney disease, an immune system disease, a condition whose treatment reduces the immune response, or a chronic neurological or neuromuscular disease.
