# Supplementary material for: Effectiveness of Pandemic and Seasonal Influenza Vaccines in Preventing Laboratory-Confirmed Influenza in Adults: A Clinical Cohort Study during Epidemic Seasons 2009–2010 and 2010–2011 in Finland
Source: PLoS One. 2014 Sep 29;9(9):e108538. doi: 10.1371/journal.pone.0108538 (PMC4180439; doi:10.1371/journal.pone.0108538)
Supplement: Table S2 — Background data of the study participants. (DOCX) [file pone.0108538.s002.docx]

**Table S2. Background data of all participants enrolled in the study in 2009-10, participants included in the analysis during the epidemic season 2010-11** **and residents of Tampere city**

|  | **Participants enrolled in 2009-10 N(%)** | **Participants included in the analysis in 2010-11 N(%)** | **Residents of Tampere city aged 18-75 years in 2010 N(%)** |
| --- | --- | --- | --- |
| All | 3815 | 2276 | 161446 |
| Age, years^1^ |  |  |  |
| 18-24 | 279 (8) | 85 (4) | 25841 (16) |
| 25-49 | 1407 (40) | 793 (35) | 76107 (47) |
| 50-64 | 1739 (49) | 1158 (51) | 40585 (25) |
| 65-75 | 93 (3) | 240 (11) | 18913 (12) |
| Females | 2280 (65) | 1473 (65) | 82 102 (51) |
| Pregnant^2^ | 45 (2) | 23 (2) | - |
| Medical target group for vaccination^3^ | 563 (16) | 417 (18) | - |
| Target group for vaccination because of occupation at start of the follow-up 2009-10**^4^** | 361 (10) | 206 (9) | - |
| Vaccinated with Pandemrix® 2009-10**^5^** | 2263 (64) | 1496 (66) | 60273 (37) |

**^1)^** Participants enrolled in the study in 2009-10 and participants included in the analysis during the epidemic season 2010-11, age at start of the follow up. Residents of Tampere, age in the beginning of the year 2010, derived from Statistics Finland.

**^2)^** Pregnant at enrolment, No (% of women). In 2009-10, the information on pregnancy was not known for 5 participants vaccinated and for 2 not vaccinated with Pandemrix®. In 2010-11 the information on the pregnancy was not available for 2 women vaccinated with both Pandemrix® and TIV and for one unvaccinated woman.

**^3)^** Individuals with at least one of the following underlying medical conditions: a heart or lung disease requiring regular medication, a metabolic disease, chronic liver failure or chronic kidney disease, an immune system disease, a condition whose treatment reduces the immune response, or a chronic neurological or neuromuscular disease. The information on medical condition was asked at enrollment in 2009-10 and it was updated at start of the follow-up in 2010-11.

**^4)^** Frontline health and social care workers treating and caring for infected patients or patients exposed to infections, including ambulance staff, and pharmacy staff attending to customers. The information on occupational risk group was not known for 3 participants vaccinated and for 2 not vaccinated with Pandemrix®. The information on occupation was asked at enrollment in 2009-10 only and it was not updated at start of the follow-up in 2010-11.

**^5)^** The vaccination status of the study participants was determined with information recorded in the electronic medical records of the health center of Tampere city or the vaccinator and additional information on received vaccination obtained credibly from the subjects through the study questionnaires. The vaccination coverage in residents of Tampere city (age 18-75) was derived from the national vaccination register
