# Supplementary material for: Effectiveness of Pandemic and Seasonal Influenza Vaccines in Preventing Laboratory-Confirmed Influenza in Adults: A Clinical Cohort Study during Epidemic Seasons 2009–2010 and 2010–2011 in Finland
Source: PLoS One. 2014 Sep 29;9(9):e108538. doi: 10.1371/journal.pone.0108538 (PMC4180439; doi:10.1371/journal.pone.0108538)
Supplement: Protocol S1 — Study protocol 2009–10. (PDF) [file pone.0108538.s003.pdf]

TERVEYDEN JA
HYVINVOINNIN LAITOS
Institutet för hälsa och välfärd

# Evaluation of a vaccination campaign with A(H1N1)v pandemic vaccines: a prospective cohort study

Study protocol: AH1N1-483-09THL, EudraCT 2009-015700-26

A(H1N1)v- pandemiarokotuskampanjan vaikutukset
Pandemiarokotuskampanjatutkimus

Alatutkimus: A(H1N1)v-immunogeenisuustutkimus

National Institute for Health and Welfare (THL), Finland

Principal Investigator Terhi Kilpi

Table of contents

1 BACKGROUND......4
2 OBJECTIVES......5
2.1 PRIMARY OBJECTIVE......5
2.2 SECONDARY OBJECTIVES......5
3 STUDY DESIGN......5
4 STUDY POPULATION......6
4.1 ELIGIBILITY CRITERIA......6
*4.1.1 Inclusion criteria......6*
*4.1.2 Exclusion criteria......7*
5 DEFINITIONS......7
5.1 A(H1N1)V-INFLUENZA LIKE ILLNESS (ILI)......7
5.2 LABORATORY CONFIRMATION OF INFLUENZA A(H1N1)V VIRUS INFECTIONS......8
5.3 VACCINATED PERSONS......8
6 VACCINATIONS......8
7 ENROLMENT......9
7.1 THE COHORT FOR ASSESSING THE EFFECTIVENESS AND SAFETY OF THE VACCINE (TOTAL STUDY COHORT).9
7.2 THE SUBCOHORT FOR ASSESSING THE IMMUNOGENICITY AND SAFETY OF THE VACCINE (IMMUNOGENICITY COHORT)......9
8 STUDY OUTCOME ASSESSMENT...... 10
8.1 ASSESSMENT OF EFFECTIVENESS...... 10
8.1.1 ASSESSMENT OF THE SEVERITY OF LABORATORY-CONFIRMED A(H1N1)V PANDEMIC INFLUENZA CASES 11
8.2 ASSESSMENT OF IMMUNOGENICITY...... 11
8.3 ASSESSMENT OF SAFETY...... 11
8.3.1 SAFETY OUTCOME EVENTS...... 12
8.3.2 OUTCOME EVENTS FROM THE HEALTH CENTRE AND HOSPITAL REGISTERS...... 12
8.3.3 OUTCOME EVENTS SPONTANEOUSLY REPORTED TO THE REGISTER OF ADVERSE REACTIONS FOLLOWING IMMUNISATION...... 13
8.3.4 OUTCOME EVENTS REPORTED BY THE PARTICIPANTS OF THE IMMUNOGENICITY COHORT...... 13
9 SERIOUS ADVERSE EVENTS (SAE) REPORTING...... 14
9.1 DEFINITION OF SAE...... 14
9.2 SERIOUS ADVERSE EVENTS AFTER A(H1N1)V VACCINATION...... 15
9.3 ASSESSMENT OF CAUSALITY...... 15
9.4 FOLLOW-UP OF SERIOUS ADVERSE EVENTS AND ASSESSMENT OF OUTCOME...... 16

9.5 TREATMENT OF SERIOUS ADVERSE EVENTS ..... 16
9.6 REGULATORY REPORTING REQUIREMENTS FOR SERIOUS ADVERSE EVENTS ..... 16
**10 LABORATORY PROCEDURES ..... 17**
10.1 LABORATORY METHODS FOR ASSESSING THE IMMUNOGENICITY OF THE A(H1N1)V VACCINE ..... 17
10.2 LABORATORY METHODS FOR IDENTIFYING OF LABORATORY-CONFIRMED INFLUENZA A(H1N1)V CASES . 18
10.2.1 NASAL AND THROAT SWAB SAMPLES ..... 18
10.2.2 BLOOD SAMPLES FOR ADDITIONAL INFORMATION ON LABORATORY-CONFIRMED A(H1N1)V INFLUENZAWAB SAMPLES ..... 19
**11 STATISTICAL METHODS ..... 19**
11.1 ENDPOINTS ..... 19
11.2 SAMPLE SIZE CONSIDERATIONS ..... 19
11.3 STATISTICAL METHODS FOR ANALYSES ..... 20
**12 DATA COLLECTION AND MANAGEMENT ..... 20**
12.3 QUALITY CONTROL ..... 21
12.4 DISCONTINUATION ..... 21
12.5 ARCHIVING ..... 22
**13 INSTITUTIONS AND INVESTIGATORS ..... 22**
**14 ETHICAL CONSIDERATIONS ..... 23**
**15 TIME SCHEDULE OF THE STUDY ..... 24**
**16 PUBLICATION OF THE RESULTS ..... 24**
**17 REFERENCES ..... 24**

# 1 Background

Pandemic influenza A viruses have caused significant morbidity and mortality during the last century (1). In April 2009 a new influenza A virus started to circulate in North America causing upper respiratory tract infections. Initially, in Mexico the mortality to this virus appeared to be relatively high (ca. 2%), but based on wide-spread diagnostic activity in all countries around the world the disease resembles more a severe seasonal influenza A virus infection. Molecular characterization of the virus by the scientists at U.S. Centers for Disease Control and Prevention (CDC) revealed that the circulating virus was a previously unrecognised strain of subtype H1N1 influenza A virus in which two polymerase genes (PA and PB2) were originating from North American avian influenza A viruses, one polymerase gene (PB1) from human seasonal influenza A virus and three genes from North American (hemagglutinin, nucleoprotein, NS protein) and two genes (neuraminidase, M protein) from Eurasian swine influenza A viruses (2). The virus likely developed in swine from a triple reassortant swine influenza A virus that has been circulating in North America (3) and another swine virus that simultaneously infected pigs. The new influenza A/H1N1 swine-lineage (swl) virus is genetically and antigenically different from past human seasonal influenza A viruses. There is approximately only 79% and 81% amino acid identity between the new A(H1N1)v (California strains) and H1N1 vaccine (Brisbane strain; H1N1 component of the 2009/2010 seasonal influenza vaccine) virus hemagglutinin and neuraminidase genes, respectively. It is thus expected that humans do not have significant pre-existing immunity against the new virus making virtually the whole world population susceptible for the disease. Rapid identification of the new influenza A(H1N1)v virus and the availability of many seed virus stocks for vaccine manufacturers has enabled a rapid development and production of vaccines against the new influenza A(H1N1)v virus. First vaccines are ready for clinical testing and many European governments have made an advance purchase agreement to buy A(H1N1)v vaccines either for the whole population or for defined priority groups for vaccination.

Finland has made an agreement to buy one dose of A(H1N1)v vaccine for the whole population and negotiates of a second dose. The likely availability of the first doses of the vaccine will enable the vaccination of at least some parts of population during the

last months of the year 2009. The decision of the vaccination strategy will be done, when enough knowledge is available of the epidemiological and clinical features of the disease. If mass vaccinations will start with a new vaccine, which can not be evaluated as thoroughly as other new vaccines, it is essential to have a plan in place to get information of the immunogenicity, safety and clinical effectiveness of the vaccine as rapidly as possible.

# 2 Objectives

## 2.1 Primary objective

- To determine the effectiveness of an A(H1N1)v influenza vaccination in preventing the first episode of laboratory-confirmed infection with the novel, pandemic influenza A(H1N1)v virus among community-dwelling and recently vaccinated adults as compared to unvaccinated adults from a cohort of at least 4,000 persons

## 2.2 Secondary objectives

- To assess the safety for 6 months after vaccination with the A(H1N1)v vaccine during the follow up;
- To determine humoral and cellular immune responses to one or two doses of the A(H1N1)v vaccine in 200 adults aged 18 to 75 years
- To evaluate the incidence, severity and possible complications of laboratory-confirmed infection with the novel A(H1N1)v influenza virus;
- To explore the effectiveness of the A(H1N1)vl vaccine in subgroups stratified by age

# 3 Study design

In case that the government recommends vaccination with pandemic A(H1N1)v vaccine, it is no longer ethical to conduct randomized trials. Therefore, a prospective observational cohort study is the most reasonable alternative in which the incidence rates of A(H1N1)v disease in exposed (i.e. vaccinated) and unexposed (i.e. unvaccinated) adults are compared (the total study cohort). This cohort design also allows assessment of the immunogenicity of the A(H1N1)v vaccine among a subgroup

of vaccinated adults (the immunogenicity cohort). Collaboration will be established with the publich health care settings providing primary, secondary and tertiary health care to persons in the study cohort for follow-up of the safety of the vaccination and severity of the A(H1N1)v influenza cases. In addition, existing medical registers will be used to follow-up of the safety and severity outcomes. In order to address the study objectives in the most optimal way, a flexible approach to study design, target group of enrollment, and the length of follow-up is required, so that changes can be made in response to accumulating information. Since no vaccine intervention is offered in the frame of this study, study enrollment procedure need to follow closely the vaccine delivery schedules, mass vaccination recommendations, as well as vaccine uptake among those who will be offered the vaccine as part of the national vaccination campaign. In addition, uncertainty regarding the peak of epidemics provide further complications, which necessitates the possibility for rapid re-evaluation of the study cohort enrollment plans and the length of follow-up, as is customary in these types of observational studies. According to the current available information, the study period of data collection is envisiaged from September-October 2009 until the first wave of A(H1N1)v influenza is considered to be over in the region, presumably by the end of April 2010.

# 4 Study population

The subjects will be enrolled from the population assigned to use the services of Tampere health centre.

## 4.1 Eligibility criteria

### 4.1.1 Inclusion criteria

- Full legal competence;
- Written informed consent obtained;
- Assigned to use the services of Tampere health centre and community-dwelling;
- At least 18 and no more than 75 years old, inclusive;
- Belongs to the target group of A(H1N1)v vaccination in the region during the pandemic vaccination campaign;

- Able to communicate fluently in Finnish or Swedish
- Able to adhere to all protocol required study procedures without any special burden or risk, as judged by the investigator or designate.

#### 4.1.2Exclusion criteria

- For the total study cohort, no specific exclusion criteria will be applied;
- For the subgroup for follow-up of immunogenicity of the vaccine (immunogenicity cohort), exclusion criteria comprise:
    - previous severe allergic reaction to influenza vaccines or known severe allergy to the ingredients of the vaccine
    - previous severe allergic reaction to eggs
    - significant immunological disorder

Participation in the study will be offered to subjects defined by the inclusion and exclusion criteria, until 4,000 subjects have been enrolled. A random sample is preferred, but the sample may be weighted by taking into account the priorisation of the national A(H1N1)v vaccination campaign and/or vaccine uptake. Participation in the immunogenicity cohort will be offered to participants of the total study cohort who intend to take the A(H1N1)v vaccine during the vaccination campaign according to the national recommendations. In total, 200 individuals will be recruited for blood sampling and follow-up of immunogenicity and safety of the vaccine.

# 5 Definitions

## 5.1 A(H1N1)v-influenza like illness (ILI)

A(H1N1)v-influenza like illness (ILI) is defined as sudden onset of the following self-reported clinical signs and symptoms:

- fever ($\geq$ 38 °C) and at least one sign or symptom of acute respiratory infection or
- pneumonia diagnosed by a physician

### 5.2 Laboratory confirmation of influenza A(H1N1)v virus infections

Cases of laboratory confirmed A(H1N1)v influenza are defined as events, where an individual belonging to the effectiveness and safety cohort and presenting with ILI defined in chapter 5.1. has A(H1N1)v influenza virus identified from a combined nasal and throat swab specimen obtained within five days after onset of the symptoms by methods described in chapter 10.2.1

- at least a 4-fold increase in A(H1N1)v influenza-specific antibody titers in serum specimens between the acute phase (0 to 5 days after onset of the symptoms) and convalescent phase (2-3 weeks after the acute phase serum sample) serum specimens will be used as additional information, if the person is unvaccinated or if at least 4 weeks have elapsed since receiving the last dose of A(H1N1)v vaccine at time of the acute phase sampling, but this is not a criteria for defining cases of laboratory confirmed influenza.

### 5.3 Vaccinated persons

The individual is defined as vaccinated if he/she has received at least one dose of A(H1N1)v vaccine, irrespective how much time has elapsed since the vaccination.

# 6 Vaccinations

It is anticipated that an A(H1N1)v vaccination campaign is conducted in the study area during the study period. The study subjects represent the target population of such a vaccination campaign and will thereby be offered at least one dose of A(H1N1)v vaccine during the study period by Tampere health centre. An agreement will be made with the health centre on recording the vaccinations individually in the electronic medical records of the health centre or into another database even during mass vaccinations.

# 7 Enrolment

## 7.1 The cohort for assessing the effectiveness and safety of the vaccine (total study cohort)

Individuals in the eligible population will be invited to participate in the study, e.g. by sending invitation letters home and by announcing in media, workplaces and/or relevant health care settings until 4,000 subjects fulfilling the eligibility criteria have been enrolled to the effectiveness cohort.

All individuals who volunteer to participate in the study will be invited to a baseline study visit. During this visit the study design and procedures are explained to the subjects, the eligibility will be assessed and a written informed consent will be obtained, including permission to collect of nasal and throat swabs and paired blood samples in case of acute ILI and permission to collect health information on baseline and outcome medical conditions and vaccinations from existing medical registers and medical records. In addition, subjects are requested to comply with weekly contacts by the study staff by SMS, email or phone call.

At the enrolment visit, baseline data and self-reported medical history, including demographics, occupation, co-morbidities, medication, vaccinations and pregnany are collected.

## 7.2 The subcohort for assessing the immunogenicity and safety of the vaccine (immunogenicity cohort)

A subgroup of 200 individuals will be recruited to participate in the cohort for assessing the immunogenicity and safety of the vaccine. Participation is offered to those persons who participate the main study (total study cohort) and intend to take the A(H1N1)v vaccine during the vaccination campaign according to the national recommendations for the population. The design and procedures for following the immunogenicity and safety of the vaccines will be explained to the subject and the eligibility will be assessed at the baseline visit. For persons who volunteer to participate in the immunogenicity study, a study visit will be arranged immediately before the first vaccination. During this visit the baseline information will be updated, the eligibility to the immunogenicity study will be reassessed, and a specific written informed consent will be obtained, including pre- and

post-vaccination blood samples for measuring immunogenicity of the vaccine. The consent also comprises reporting serious and important adverse events as well as adverse events with special interest (8.3.1) for a period of 6 months after the last vaccination, and permission to verify the diagnoses in medical records.

# 8 Study outcome assessment

## 8.1 Assessment of effectiveness

At enrolment, all 4,000 subjects participating in the effectiveness and safety cohort will be instructed to detect signs of ILI, defined in chapter 5.1. If ILI is present, the participants are instructed to contact the study staff immediately to have nasal and throat swab specimens and a blood sample taken as soon as possible, preferably within 3 days and at least within 5 days after the start of symptoms. Subjects are asked to fill in a diary in case of ILI to characterize the start of symptoms and clinical features of the disease In addition, all study subjects will be contacted weekly by SMS, if possible, or by email or phone call in order to query any current or previous symptoms of ILI.

In case of ILI, an acute phase visit will be arranged 0 to 5 days after the onset of symptoms, either at the home of the subject or in the premises of collaborating health care settings or at a special study clinic established for the study. Nasal and throat swabs will be obtained by a qualified study nurse or physician, to define the cases of laboratory confirmed A(H1N1)v influenza (5.2). In addition, 10 ml of venous blood will be drawn at the acute phase. Clinical features of ILI will be recorded using the diary as an aid and a convalescent visit will be scheduled 2-3 weeks after the acute phase visit. Another diary will be given for follow up of the course of the disease. At the convalescent visit, 10 ml of venous blood will be drawn for measurement of the increase in A(H1N1)v virus specific antibody levels, and further information of the disease will be collected using the diary as an aid. After the first occurrence of laboratory-confirmed A(H1N1)v influenza case, no subsequent individual follow-up will be performed for the effectiveness outcome, i.e. for ILI.

The information regarding A(H1N1)v vaccination will be obtained from the health care data system of Tampere health centre.

### 8.1.1 Assessment of the severity of laboratory-confirmed A(H1N1)v pandemic influenza cases

Information on potential missed cases will be collected by weekly contacts to study participants and by collecting the positive laboratory test results for A(H1N1)v virus from the data bases of the Tampere city and TAUH and from the Communicable Disease Register of THL.

To evaluate the severity of the disease, medical records of Tampere city and TAUH will be visited for cases defined in chapter 5.2. The clinical features of the disease and and medical consultations will be asked at the acute and convalescence phase visits during ILI using the diary as an aid. If needed and possible, medical record will be requested from other than collaborating health care settings as well. Additional information on infections related to A(H1N1)v influenza may be requested from the Communicable Disease Register (e.g. invasive bacterial diseases).

## 8.2 Assessment of immunogenicity

From the subgroup of the 200 persons participating in the cohort for assessing the immunogenicity of the A(H1N1)v vaccine, 60 ml of venous blood will be drawn at the day of (and before) the first vaccination. At a visit scheduled at 4 weeks after vaccination, 60 ml of venous blood will be drawn for assessment of humoral and cellular immunity. Immunogenicity will be assessed as the fold-increase between pre- and post-vaccination immune responses. If the national vaccination strategy will include two doses of A(H1N1)v vaccine, similar pre-vaccination blood sample (60 ml) will be drawn at the day of the second vaccination and a post-vaccination blood sample (60 ml) will be obtained 4 weeks after the second vaccination, for assessing the humoral and cellular immune reaction to the second vaccine dose. A blood sample (60 ml) for assessing the long term immunity level is scheduled to be obtained 6 months after the administration of the last vaccine dose.

## 8.3 Assessment of safety

The safety of the A(H1N1)v vaccine will be followed from health care registers in the total study cohort of 4,000 study subjects and in the immunogenicity cohort, by interview

at the study visits. Baseline medical conditions and medications are recorded at the time of enrolment, and permission to verify the diagnosis from medical records is included in the consent. Permission to verify background data from medical records will also be requested.

### 8.3.1Safety outcome events

The list of pre-defined safety outcomes will be adapted from the list of adverse events recommended by European Medicines Agency (EMEA) to be followed during pandemic vaccination (4,5). They comprise:

Serious (unexpected) adverse reactions

Adverse events of special interest (AESI), e.g.:

- severe allergic reactions,
- convulsions,
- neuritis,
- encephalitis,
- vasculitis,
- Guillain-Barré Syndrome,
- other demyelinating disorders,
- Bell's palsy,
- vaccination failures

The list may be flexibly adapted according to the information arising nationally and internationally, e.g. by spontaneous reporting of adverse events, experience from vaccination and studies or new recommendations of relevant authorities.

### 8.3.2 Outcome events from the health centre and hospital registers

Data recorded in the electronic health care data systems during the visits of the study participants to Tampere health centre, Tampere city hospital and Tampere University hospital (TAUH) during the study period will be collected. These data comprise e.g. reasons of the visits, diagnoses made at them, referrals to hospitals, policlinics and specialists, hospitalisations, emergency room visits and possible cases of death, laboratory records etc. Individual medical records will be visited for additional information, if needed.

### 8.3.3 Outcome events spontaneously reported to the register of adverse reactions following immunisation

Finnish health care professionals are responsible for reporting serious and/or unexpected adverse reactions following immunisation (AEFI) to Unit of Vaccination Programme (ROHY) of the National Institute for Health and Welfare (THL) by using a form (AEFI form) designed for this purpose (decree no. 421/2004 given by the Ministry of Social Affairs and Health). In addition, a specific form with pre-defined events with special interest will be established for reporting adverse events after vaccination with A(H1N1)v vaccines. ROHY enters information on these reports to an electronic register of adverse reactions following immunisation (AEFI register), which will be an additional method for collecting safety data during the study period.

The healthcare professional will send the AEFI report form by mail or by fax to ROHY without undue delay. In urgent cases the reporting can be also done by phone. Additional or follow-up information relating to the initial suspected adverse event report is also to be completed and submitted with an AEFI form immediately.

If ROHY receives a report of AEFI considered to fulfill the criteria of a serious adverse event (SAE, definition 9.1) after A(H1N1)v vaccination of a participant of the total study cohort, the study staff will be notified by phone immediately and the report will be sent by fax to the investigator and/or designate of the study as soon as possible. The non serious adverse events and AESI cases will be reported to the investigators and/or designate on regular basis without expedition.

### 8.3.4 Outcome events reported by the participants of the immunogenicity cohort

The 200 subjects participating in the immunogenicity cohort are asked to report the occurrence of serious and important adverse events as well as AESI occurring during the study period up to 6 months after the last vaccination. For this reason, a diary will be given at the first study visit. The diagnoses and details of the events will be verified from medical records, if needed and possible.

# 9 Serious adverse events (SAE) reporting

## 9.1 Definition of SAE

An adverse event is any untoward medical occurrence in a patient or clinical investigation subject administered a pharmaceutical product and which does not necessarily have a causal relationship with this treatment. An adverse event (AE) can therefore be any unfavourable and unintended sign (including an abnormal laboratory finding), symptom, or disease temporally associated with the use of a medicinal product, whether or not related to the medicinal product.

An adverse event is defined as 'serious' when it meets one of the following criteria:

1. results in death,
2. is life-threatening,
   - The term 'life-threatening' in the definition of 'serious' refers to an event in which the subject was at risk of death at the time of the event. It does not refer to an event which hypothetically might have caused death, if it were more severe.
3. requires hospitalisation or prolongation of existing hospitalisation,
   - Hospitalisation signifies that the subject has been detained (or involving at least an overnight stay) at the hospital or emergency ward for observation and/or treatment that would not have been appropriate in the physician.s office or outpatient setting. If a complication that occur during hospitalisation prolongs hospitalisation or fulfils any other serious criteria, the event is serious. When in doubt as to whether hospitalisation occurred or was necessary, the event should be considered serious. Hospitalisation for elective treatment of a pre-existing condition that did not worsen from baseline is not considered a SAE.
4. results in persistent or significant disability/incapacity
5. is a congenital anomaly/birth defect in the offspring of a study subject.

In addition, medical or scientific judgement will be exercised in deciding whether an adverse event is serious in other situations. Important medical events that may not be immediately life-threatening or result in death or hospitalisation but may jeopardize the subject or may require medical or surgical intervention to prevent one of the other outcomes listed in the above definition, will also be considered serious. Examples of such events are invasive or malignant cancers, intensive treatment in an emergency

room or at home for allergic bronchospasm, blood dyscrasias or convulsions that do not result in hospitalisation.

### 9.2 Serious adverse events after A(H1N1)v vaccination

All SAEs following A(H1N1)v vaccination of the participants of the total study cohort reported to the AEFI register during the clinical follow-up and 6 months thereafter, as well as SAEs reported by the participants of the immunogenicity cohort within 6 months after the last vaccination will be collected. When the investigator and/or designate becomes aware of a SAE, all relevant available documentation (e.g., hospital progress notes, laboratory, and diagnostics reports) relative to the event will be review and all relevant information will be recorded in the study database. The investigator and/or designate will attempt to establish a diagnosis of the event based on signs, symptoms, and/or other clinical information. In such cases, the diagnosis will be documented as the SAE and not the individual signs/symptoms.

### 9.3 Assessment of causality

The investigator and/or designate will assess the relationship between the A(H1N1)v vaccination and the occurrence of each SAE defined in chapter 9.1. Alternative causes, such as natural history of the underlying diseases, concomitant therapy, other risk factors and the temporal relationship of the event to the vaccination will be considered and investigated. The investigator and/or designate will follow the recommendations of WHO, in the determination of his/her assessment.

SAEs will be examined by the investigator and/or designate to the extent to be able to determine all contributing factors applicable. Other possible contributors may include e.g. medical history, other medication, protocol required procedure, other procedure not required by the protocol, lack of efficacy of the vaccine, erroneous administration or other cause.

The causal relationship between the A/H1N1 vaccination and the event will be assessed as

- unrelated; the causal relation is not plausible and/or there is another clear cause for the event

- unlikely; the causal relatioship can not be ruled out, but there is another, more probable cause for the event
- possible; the causal relationship is possible, but there is another, equally probable cause for the event
- probable; causality is plausible and there is no other as probable cause for the event

### 9.4 Follow-up of serious adverse events and assessment of outcome

After identifying SAEs defined in chapter 9.2 the investigator and/or designate will follow up each subject until the event has resolved, subsided, stabilised, disappeared, the event is otherwise explained, or the subject is lost to follow-up. Relevant additional information will be updated in the study database.

### 9.5 Treatment of serious adverse events

Treatment of any SAE or other adverse events of the study participants is at the sole discretion of the physician responsible for the person's medical care and according to current good medical practice.

### 9.6 Regulatory reporting requirements for serious adverse events

According to the national legislation, THL will report to the National Agengy of Medicines and the ethics committee of PSHP all SAE cases defined in chapter 9.2. for which the causal relationship with the A(H1N1)v vaccine can not be ruled out and which are unexpected according to the investigator's broschure within time limits defined in the regulation of the National Agengy of Medicines (LL Dnro 2740/0.5.1./2007 ). Once a year, THL will provide the National Agency for Medicines and the ethics committee of PSHP with a list of all SAE cases defined in chapter 9.2. with potential causal relationship to the A(H1N1)v vaccine which have occurred to the particiapants of the effectiveness cohort during the SAE detecting period, defined to end 6 months after the end of the clinical follow up of the total study cohort, and for the events reported by the participants of the immunogenicity cohort, when 6 months has elapsed since the date of the last vaccination.

The occurrence of serious adverse events and AESI recognised in the register data collected from the health care data systems of Tampere city and TAUH is a result variable of this large cohort study. Recognising, analysing and reporting individual SAE cases from register data only or analysing suspected cases will not be possible in the limits of resources of this study. Summaries of adverse reactions relating to these safety result variables will be supplied to the National Agency for Medicines and ethics committee in the study report.

# 10 Laboratory procedures

## 10.1 Laboratory methods for assessing the immunogenicity of the A(H1N1)v vaccine

Blood specimens for assessing immunogenicity of the A(H1N1)v vaccine will be processed for white blood cell isolation (from 50 ml of blood) and obtaining serum specimens (from 10 ml of blood). The samples for cellular immunity assays will be sent to the Virus Infection Unit (ROVI) of THL, Department of Vaccination, where the peripheral blood mononuclear cells (PBMC) will be isolated and stored at -130 °C or colder, and cell-mediated immune responses will be analyzed simultaneously for all 3-5 specimens obtained from the same subject according to the instructions of ROVI. Serum specimens will be stored at -20 °C or colder and humoral immune responses will also be analyzed for all 3-5 specimens simultaneously.

Humoral immunity against the vaccine virus (likely A/California/7/2009 H1N1v or equivalent) will be studied using standard hemagglutination inhibition (HI) and neutralization (NT) tests (6). Study viruses include the wild type virus that is used for the vaccine for studying the homologous response and most recent swine-origin lineage viruses that have been isolated in the fall 2009 as well as other recent H1N1 (Brisbane/59-like viruses) and H3N2 (Brisbane/10-like viruses) viruses. All viruses required for antibody determination are either already available or will be obtained (new influenza A(H1N1)v isolates) prior to the study.

## 10.2 Laboratory methods for identifying of laboratory-confirmed influenza A(H1N1)v cases

### 10.2.1 Nasal and throat swab samples

Viruses are intracellular pathogens. In order to obtain a good diagnostic sample for viral studies it must be attempted to collect as many epithelial cells as possible from the nostrils and the throat without causing excessive discomfort to the patient. To achieve maximal sensitivity, both nostrils and the throat must be swabbed. Nasal and throat swab specimens will be obtained from individuals within 5 days after onset of ILI symptoms defined in chapter 5.1.

To obtain the nasal sample, a preferably flocked swab will be inserted approximately 3 – 4 cm into the first nostril, left in place for a few seconds, then slowly withdrawn with a rotating motion in order to trap as many epithelial cells as possible. If possible, the same swab will be used to sample also the other nostril. The swab will be broken into the tube containing transport medium. The throat, the tonsils, the uvula, and the posterior wall of the pharynx will be sampled using a fresh swab. This swab will be broken into the same tube containing the nasal swab. The sample will be placed in a refrigerator (temperature $4^{o}$ C) for short-term storage (24 – 48 hours), and/or in a freezer ($\leq$ $-60^{o}$ C) for extended storage until analysed. If needed, the samples will be sent to the laboratory by overnight mail in a small styrofoam box containing cool-cushions.

In ROVI, A(H1N1)v influenza viruses identified by reverse transcription polymerase chain reaction (RT-PCR) method and, if needed, virus culture. For differential diagnostics and potential assessment for assiociations, influenza A and B as well as respiratory syncytial virus and other visuses causing respiratory will be identified from the combined nasal-throat swab specimens. Furthermore, typing and subtyping may be performed using instructions of ROVI. Resistance to oseltamivir may be determined from selected samples.

RT-PCR methods for detecting influenza A (and B) virus-specific RNAs are readily available in the laboratory. Virus Infection Unit at THL functions as a National Influenza Center (NIC) and the unit carries out national surveillance work for the identification of circulating influenza virus strains in Finland. Approximately 1,000 nasopharyngeal specimens/year are screened and 150-200 influenza viruses identified and cultured

each year. Influenza A virus-specific RT-PCR is based on general identification of influenza A viruses by matrix (M1) gene-specific primers that identify all influenza A viruses independent of their H or N gene subtype. Virus Infection Unit has also developed swine-origin pandemic influenza A(H1N1)v specific RT-PCR assay based on H1 and NS1 gene specific primers that reliably enable the detection of swine-origin A(H1N1)v virus infections. The assay was successfully used to determine the first influenza A(H1N1)v infection cases in Finland in early May, 2009. Thus the methodology for the rapid and specific influenza A virus diagnostics, for isolation and further characterization of influenza viruses are well established in the laboratory.

#### 10.2.2 Blood samples for additional information on laboratory-confirmed A(H1N1)v influenzawab samples

Of blood samples (á 10 ml) obtained at acute and convalescent phases of ILI, serum specimens will be prepared and stored at -20 °C or colder until analysed. The antibody titers will be measured using standard hemagglutination inhibition and neutralization tests (6).

# 11 Statistical methods

## 11.1 Endpoints

The study endpoints for effectiveness, immunogenicity and safety are defined in, respectively, 5.2, 8.2 and 8.3.

## 11.2 Sample size considerations

Based on recent questionnaire data from Finland, as part of FLUMODCONT (pandemic FLU MODelling CONTainment strategies),project, the assumption is that, if offered the vaccine, 90% will take it. Hence the expected number of unvaccinated persons in the study cohort is 400. Furthermore, it is assumed that during the first wave of the A(H1N1)v pandemic, which is the planned period of follow-up in this study, 20% of the unvaccinated become infected by the A(H1N1)v virus, i.e. 80 expected cases. The assumed effectiveness of the vaccine is 70%. The study is

powered to demonstrate the effectiveness of the vaccine such that the lower level of the 95% confidence interval is above 50%, which is regarded as the level of clinical relevance for the A(H1N1)v vaccine. With a random sample size of 4,000 the power of the study is 95%. High power for the effectiveness evaluation is preferred, since taking into account potential confounders, as well as possible unbalance in the age distribution of the study cohort (see Section 3) will lower the power.

### 11.3 Statistical methods for analyses

The incidence of the laboratory-confirmed H1N1 pandemic influenza will be calculated as the number of cases divided by the person-time at risk, where person-time at risk is defined as compliance to the regular contacts. Periodic variation of incidence in calendar time will also be explored both in vaccinated and unvaccinated groups. Event-history analysis methods will be used for the evaluation of the effectiveness of the vaccine, where the risk of acquiring A(H1N1)v-influenza will be compared between the vaccinated/unvaccinated states, by accounting for calendar time and other potential confounders. Vaccine effectiveness will be calculated as 1- relative risk. For the vaccine adverse events, case-series method will be used for comparing the incidences between risk and non-risk periods. Descriptive analysis will be conducted for the immunogenicity cohort, such as calculation of geometric mean titres and percentages over pre-defined thresholds. Due to the nature of the study (see Section 3), other descriptive analyses and tests of hypotheses may also be carried out but the choice of subsequent hypotheses will be data dependent

## 12 Data collection and management

### 12.1 Data entry

Clinical data collected at the study sites will be collected on paper CRFs or entered electronically during the visits. Software allowing direct validation of entry using defined entry criteria for each field, crosschecks of various fields and control for missing data will be used. A paper back-up system will be available. The data may be optionally entered in an off-line mode, as the data entry may take place in various study sites. The electronic data will be incorporated into the study database daily using established

systems for data transportation.

Bacteriologic and laboratory data will be entered manually after completion of the assays using the same software as for clinical data. Appropriate back-up files are maintained continuously.

### 12.2 Personal data and confidentiality

At enrollment, each patient will be assigned an individual subject identification (ID) number. Subjects' full identification information will be recorded in the database. The study related personal and other confidential data will be managed confidentially, according to the national regulations and internal rules and guidelines of THL.

### 12.3 Quality control

THL will perform internal quality control and monitoring. This will include education and training prior to study start, writing of study-specific SOPs and close follow-up of the study during the clinical phase. Study documentation and records retention will be performed according to the archiving rules of ICH and THL.

The compliance to the ICH guidelines for Good Clinical Practice and other quality requirements will be achieved by education of the study staff, according to the research tradition of the department, guidelines of the the handbook ' Good research practice in the National Public Health Institute' as well as other guidelines and internal regulations of the institute.

### 12.4 Discontinuation

If the subject withdraws his/her consent, the collection of data will be stopped. As main rule, the data recorded before the discontinuation of a subject will not be removed from the study database.

### 12.5 Archiving

The investigators' files and other study related documents are stored in locked cabinets in the study centre during the clinical phase, data resolution and analysis, as long as needed. Thereafter the documents are transposed to the final archive facilities according to the institutional Archiving Guidelines and under supervision of the Senior Archivist of the institute. An archive index is maintained to record all research files that have been entered into the archive or destroyed. In general, all original research material generated at THL is retained permanently according to the national Archives Act (831/1994). THL will arrange access to the study documents during the follow up and after archiving and collaborate with the relevant persons authorised to perform audits.

## 13 Institutions and investigators

Sponsor: National Institute for Health and Welfare (THL)

Principal investigators:

Principal investigator: T. Kilpi, M.D., Ph.D., Paediatrician, Head of Department of vaccination and immune protection, THL

Other investigators in Finland:

Jukka Jokinen, Ph.D., Statistician, THL

Tuija Leino, M.D., Ph.D., Senior researcher, THL

Ritva Syrjänen, M.D., Researcher, Clinical coordinator/study physician, THL

Jonas Sundman, Data management, THL

Laboratory analyses:

Ilkka Julkunen, M.D. ,Ph.D., Professor, Head

Thedi Ziegler, Ph.D., Laboratory Head

Mari Strengell, Ph.D., Research scientist

Esa Rönkkö, MSc., Research scientist

Niina Ikonen, MSc., Research scientist

Riitta Santanen, Lab. Technician

Anja Willberg, Lab. Technician

Virus Infection Unit, Department of Vaccination and Immune Protection

National Institute for Health and Welfare (THL), Helsinki, Finland

## 14 Ethical considerations

The study will be conducted according to the ICH guidelines for Good Clinical Practice (GCP), the Declaration of Helsinki, local rules and regulations of the country. The study protocol will be submitted to the ethics committee of the Pirkanmaa Hospital District (PSHP) and the National Agency for Medicines. Appropriate permissions will be obtained from the clinical sites taking part in the study and institutes keeping registers. Appropriate permission to combine the register data will be applied from the Ministry of Social Affairs and Health.

Although the aim of the study is to study influenza vaccinations, the vaccines given during the study period are not an intervention introduced for the purposes of this study but part of the national vaccination strategy aiming at protecting the subjects from pandemic influenza. Thus no additional risk is created.

Written informed consent will be obtained from the subjects, separately for the effectiveness study and for the immunogenicity study. The consents include the nasal, throat and blood samplings and the use of medical register data and medical records about vaccination and relevant health outcomes.

Nasal and throat samples and blood samples to be collected in the study are generally accepted methods in clinical practice. The study procedures thus carry out minimal additional risk to the subjects as compared to normal clinical practices. The study procedures will be performed by experienced health care personnel only.

## 15 Time schedule of the study

The enrolment is estimated to start when the epidemic is considered to have started in the region or when the government has decided to start vaccination against A(H1N1)v influenza. The study period for follow-up of occurrence of laboratory-confirmed A/H1N1)v influenza cases is envisioned from September-October 2009 until the first wave of A(H1N1)v influenza is considered to be over in the region, and 30 April 2010 at latest. The study period for collecting immunogenicity data depends on availability of vaccines and will last until 6-7 months have elapsed since the last vaccination of the participants of the immunogenicity study. The follow-up of safety and severity outcome events from registers will last up to 6 months after the end of the clinical follow up. If relenvant reasons rise during the pandemic, the consent allows a long term follow up with registers up to 3 years after the start of the study. The consent will also include permission to follow up the health of children born to women who are pregnant during the follow up until the child is one year old. The laboratory analyses and statistical analysis are phased from April 2010 until the end of 2010.

## 16 Publication of the results

Results of the study will be published as collaborative articles in scientific meetings and peer-reviewed medical journals with the principal investigator (TK) deciding on the authors and order, based on the proposals made by the research teams.

## 17 References

1. Horimoto T, Kawaoka Y. Influenza: lessons from past pandemics, warnings from current incidents. Nat Rev Microbiol. 2005 3:591-600.

2. Novel Swine-Origin Influenza A (H1N1) Virus Investigation Team. Emergence of a Novel Swine-Origin Influenza A (H1N1) Virus in Humans**.** N Engl J Med 2009 0: NEJMoa0903810

3. Shinde, V., Bridges, C.B., Uyeki, T.M., ym. Triple-Reassortant Swine Influenza A (H1) in Humans in the United States, 2005-2009**.** N Engl J Med 2009 0: NEJMoa0903812

4. CHMP Recommendations for the Core Risk Management Plan for Influenza Vaccines prepared from viruses with the potential to cause a pandemic and intended for use outside of the core dossier context. EMEA/49993/2008

5. CHMP Recommendations for the Pharmacovigilance Plan as part of the Risk Management Plan to be submitted with the Marketing Authorisation Application for a Pandemic Influenza Vaccine. EMEA/359381/2009

6. Lehtoranta L, Villberg A, Santanen R, Ziegler T. A novel, colorimetric neutralization assay for measuring antibodies to influenza viruses. J Virol Methods. 2009, 159:271-276.
