# Supplementary material for: Effectiveness of Pandemic and Seasonal Influenza Vaccines in Preventing Laboratory-Confirmed Influenza in Adults: A Clinical Cohort Study during Epidemic Seasons 2009–2010 and 2010–2011 in Finland
Source: PLoS One. 2014 Sep 29;9(9):e108538. doi: 10.1371/journal.pone.0108538 (PMC4180439; doi:10.1371/journal.pone.0108538)
Supplement: Protocol S2 — Study protocol 2010–11. (PDF) [file pone.0108538.s004.pdf]

TERVEYDEN JA HYVINVOINNIN LAITOS
Institutet för hälsa och välfärd

Evaluation of the effectiveness of vaccination with 2009 H1N1 vaccines during influenza season 2010-11

2009 H1N1-influenssarokotteiden tehokkuus influenssakaudella 2010-11

Pandemiarokotuskampanjan jatkotutkimus

Study protocol: H1N1-495-10THL, EudraCT 2010-021033-30

Continuation study for the study 'Evaluation of a vaccination campaign with A(H1N1)v pandemic vaccines: a prospective cohort study', AH1N1-483-09THL, EudraCT 2009-015700-26

28.5.2010

National Institute for Health and Welfare (THL), Finland

Principal Investigator Terhi Kilpi

Table of contents

1 BACKGROUND....4
2 OBJECTIVES....5
2.1 PRIMARY OBJECTIVE....5
2.2 SECONDARY OBJECTIVES....5
3 STUDY DESIGN....5
4 STUDY POPULATION....7
4.1.1 Inclusion criteria for the interview follow-up....7
4.1.2 Inclusion criteria for the confirmation of 2009 H1N1 influenza cases....7
4.1.3 Exclusion criteria....7
5 DEFINITIONS....8
5.1 2009 H1N1 INFLUENZA LIKE ILLNESS (ILI)....8
5.2 LABORATORY CONFIRMATION OF 2009 H1N1 INFLUENZA CASES....8
5.3 VACCINATED PERSONS....8
6 VACCINATIONS....8
7 ENROLMENT....10
7.1 ENROLLMENT IN THE INTERVIEW FOLLOW-UP PHASE OF VACCINATIONS AND INFLUENZA LIKE ILLNESS....10
7.2 ENROLLMENT IN THE LABORATORY CONFIRMATION PHASE OF 2009 H1N1 INFLUENZA CASES....10
8 STUDY OUTCOME ASSESSMENT....11
8.1 ASSESSMENT OF THE VACCINATION STATUS IN THE INTERVIEW FOLLOW-UP....11
8.2 ASSESSMENT OF THE OCCURRENCE OF ILI IN THE INTERVIEW FOLLOW-UP....11
8.3 ASSESSMENT OF THE LABORATORY-CONFIRMED 2009 H1N1 INFLUENZA CASES....12
8.3.1 ASSESSMENT OF THE SEVERITY AND POSSIBLE COMPLICATIONS OF LABORATORY-CONFIRMED 2009 H1N1 PANDEMIC INFLUENZA CASES....12
8.3.2 DATA FROM THE REGISTERS OF THE HEALTH CARE CENTRE, LOCAL HOSPITALS AND THE NATIONAL INFECTIOUS DISEASE REGISTER....12
9 REPORTING OF SERIOUS ADVERSE EVENTS....13
10 LABORATORY PROCEDURES....14
10.1 LABORATORY METHODS FOR IDENTIFYING OF LABORATORY-CONFIRMED 2009 H1N1 INFLUENZA CASES 14
10.1.1 NASAL AND THROAT SWAB SAMPLES....14
10.1.2 BLOOD SAMPLES FOR ADDITIONAL INFORMATION ON ILI CASES....16
11 STATISTICAL METHODS....16
11.1 ENDPOINTS....16
11.2 SAMPLE SIZE CONSIDERATIONS....16

11.3 STATISTICAL METHODS FOR ANALYSES 17

12 DATA COLLECTION AND MANAGEMENT 17

12.3 QUALITY CONTROL 18

12.4 DISCONTINUATION 18

12.5 ARCHIVING 18

13 INSTITUTIONS AND INVESTIGATORS 19

14 ETHICAL CONSIDERATIONS 20

15 TIME SCHEDULE OF THE STUDY 20

16 PUBLICATION OF THE RESULTS 21

17 REFERENCES 21

1 Background

The pandemic caused by 2009 H1N1 influenza A virus entered Finland in May 2009. During the summer there were approximately 300 cases most of which were detected in dividuals who had contracted the disease outside Finland. From September/October onwards there were increasing numbers of domestic cases and the epidemic peaked at weeks 43 to 49. Unexpectedly, the epidemic started in Northern Finland and spread with two weeks to central and southern parts of the country. By Christmas the epidemic was virtually over in Finland and no second epidemic peak was observed during the winter months in 2010. Altogether nearly 8000 laboratory confirmed diagnoses were made in Finland. Apprimately 1500 influenza virus infected people were hospitalized, 150 people were treated at intensive care units and 44 deaths were associated with pandemic virus infection (ref 1).

The first doses of pandemic virus vaccine (Pandemrix) were received at week 42 2009 and during the subsequent weeks healths professionals, pregnant women and risk groups aged from 6 months to 65 years were vaccinated. Thereafter, healthy individuals from younger age groups followed by people over 65 years of age were vaccinated during the early months of 2010. At present vaccination coverage in Finland is approximately 50-55% and thus a great percentage of the population has still remained unvaccinated. Since there is a great number of people, who are not immune against the 2009 pandemic influenza A another epidemic peak is expected later this year of early 2011. Comprehensive virological characterization of apprimately 150 virus strains collected in Finland indicate that the virus is changing, but the rate of changes in viral hemagglutinin (HA) and neuraminidase (NA) genes does not exceed 2.2 % or 1.1%, respectively. It is thus expected that the original California/7/09 pandemic virus (ref 2) vaccine is likely to give protective immunity against the 2010/2011 season virus strains. WHO has given the recommendations for the next season influenza A virus vaccine and the seasonal influenza vaccine has the California/7/09 strain as the H1N1 vaccine component. Finland has purchased the seasonal influenza vaccine, which thus includes the pandemic 2009 H1N1 virus vaccine component, for medical risk groups, young children between 6-35 months and possibly for the health professionals and pregnant women.

The initial efficacy and immunogenicity trial carried out in Tampere between November 2009 and April 2010 failed to give relevant information for efficacy of the vaccine, since the epidemic peak in Tampere was very steep and epidemic reached Tampere area before the general population was vaccinated with the pandemic vaccine. Therefore, it is very important to continue

the initial vaccination study during the future epidemic season of 2010/2011. We thus apply permission to continue to pandemic vaccine efficacy trial.

2 Objectives

2.1 Primary objective

To determine the effectiveness of 2009 H1N1 influenza vaccination in preventing the first episode of laboratory-confirmed influenza caused by the 2009 H1N1 influenza virus or its variation during the influenza season 2010-11 among vaccinated adults as compared to unvaccinated adults participating in the study "Evaluation of a vaccination campaign with A(H1N1)v pandemic vaccines: a prospective cohort study"

2.2 Secondary objectives

- To explore the ability of different vaccination regimens to prevent 2009 H1N1 influenza;
- To explore the effectiveness of the 2009 H1N1 vaccines in subgroups stratified by age;
- To evaluate the incidence, severity and possible complications of laboratory-confirmed infection with the 2009 H1N1 influenza virus.

3 Study design

A prospective observational cohort study is the best way to evaluate the effects of large scale vaccination. Such prospective study, "Evaluation of a vaccination campaign with A(H1N1)v pandemic vaccines: a prospective cohort study, AH1N1-483-09THL" was conducted from November 2009 to April 2010 to compare the incidence rates 2009 H1N1 influenza in exposed (i.e. vaccinated) and unexposed (i.e. unvaccinated) adults. This study covered part of the first wave of the 2009 H1N1 pandemic. In the current study, the effects of 2009 H1N1 influenza vaccination will be evaluated in the same study cohort (originally 3500 participants) during the influenza season 2010-11, during which the second wave of 2009 H1N1 influenza is expected to occur.

No vaccines are administered in the study, but the participants may or may not take one or more doses of 2009 H1N1 influenza vaccines as part of the pandemic vaccination campaign, as part of the national vaccination program or as commercial products, as described in chapter 6. The participants will be asked to report all influenza vaccinations obtained at any health care setting, and the details are verified by medical records or by vaccination cards fulfilled by medical personnel, if possible. In addition, the details of vaccination will be collected from the electronic health care data system of Tampere health care centre, providing and recording all vaccinations according to the pandemic vaccination campaign and according to the national vaccination program.

After the start of an epidemic of influenza caused by the 2009 H1N1 virus, the participants will be asked to spontaneously report the occurrence of symptoms and signs of influenza like illness (ILI) defined in chapter 5.1. They are also contacted weekly by SMS or phone. The participants with symptoms and signs of ILI will be invited to a study visit, at which nasal and oral throat swab specimens will be obtained for laboratory confirmation of 2009 H1N1 influenza cases. Serum samples will be obtained at acute phase and convalescent phase 2 to 3 weeks later. The consents of the current study will include permission to visit the medical records of reported cases to find missed cases and examine the severity and possible complications of laboratory-confirmed infections with the 2009 H1N1 influenza virus. According to the protocol and consent of the original study AH1N1-483-09THL, register data on visits to the Tampere health care centre or regional hospitals as well as data from the National Infectious Disease Register of THL will be extracted. A permission to use this data for porposes of the current study will be included in the consents.

The study will comprise two phases. In the “interview follow-up” phase, the data on vaccination and occurrence of ILI cases will be collected by mail and interview by SMS and/or phone, and verified in registers and medical records, if needed. The phase in which the persons with reported symptoms or signs of ILI are invited to study visits during which laboratory samples will be obtained for identification and confirmation of 2009 H1N1 influenza cases is called “confirmation of 2009 H1N1 influenza cases” (confirmation phase).

4 Study population

Participation in the study will be offered to all participants of the cohort study “Evaluation of a vaccination campaign with A(H1N1)v pandemic vaccines: a prospective cohort study” AH1N1-483-09THL, who still live in the study area (city of Tampere) and who have complied to the SMS or phone follow-up. The eligibility criteria of the original study AH1N1-483-09THL were: full legal competence; community-dwelling; age 18 to 75 years, written informed consent obtained; able to communicate fluently in Finnish or Swedish and able to adhere to all protocol required study procedures without any special burden or risk. Originally, 3500 persons participated in the study.

4.1.1 Inclusion criteria for the interview follow-up

- Participating in the study “Evaluation of a vaccination campaign with A(H1N1)v pandemic vaccines: a prospective cohort study” (AH1N1-483-09THL);
- Assigned to use the services of Tampere health care centre;
- Written consent for the interview follow-up phase obtained by mail;
- Able to adhere to all protocol-required study procedures without any special burden or risk, as judged by the participant himself/herself

4.1.2 Inclusion criteria for the confirmation of 2009 H1N1 influenza cases

- Participating in the study interview follow-up of the current study ‘Evaluation the effectiveness of vaccination with 2009 H1N1 vaccines during influenza season 2010-11 (H1N1-495-10THL)
- Assigned to use the services of Tampere health care centre;
- Written informed consent for the confirmation phase obtained at the first study visit;
- Able to adhere to all protocol required study procedures without any special burden or risk, as judged by the investigator or designate.

4.1.3Exclusion criteria

- no specific exclusion criteria will be applied

5 Definitions

5.1 2009 H1N1 influenza like illness (ILI)

2009 H1N1 influenza like illness (ILI) is defined as sudden onset of the following self-reported clinical signs and symptoms:

- fever ($\geq$ 38 °C) and at least one sign or symptom of acute respiratory infection or
- pneumonia diagnosed by a physician

5.2 Laboratory confirmation of 2009 H1N1 influenza cases

Cases of laboratory confirmed 2009 H1N1 influenza are defined as events, where an individual belonging to the follow-up phase of the study and presenting with ILI defined in chapter 5.1. has 2009 H1N1 influenza virus identified from a combined nasal and throat swab specimen obtained within 7 days after onset of the symptoms by methods described in chapter 10.1.1

- at least a 4-fold increase in 2009 H1N1 influenza A virus-specific antibody titers in serum specimens between the acute phase (0 to 7 days after onset of the symptoms) and convalescent phase (2-3 weeks after the acute phase serum sample) serum specimens will be used as additional information, if the person is unvaccinated or if at least 4 weeks have elapsed since receiving the last dose of 2009 H1N1 vaccine at time of the acute phase sampling, but this is not a criteria for defining cases of laboratory-confirmed 2009 H1N1 influenza.

5.3 Vaccinated persons

The individual is defined as vaccinated if he/she has received at least one dose of 2009 H1N1 vaccine, irrespective how much time has elapsed since the vaccination and documentation of vaccination in the medical records of the subject.

6 Vaccinations

In Finland, one dose of the monovalent pandemic 2009 H1N1 vaccine chosen to the pandemic vaccination campaign (Pandemrix®, GlaxoSmithKline Biologicals) has been offered to the whole population. Vaccination is voluntary and free of charge. The most

active vaccination campaign was conducted in the study region Tampere between October 2009 and May 2010. During this period, the vaccination coverage in Tampere has been about 50 %. The campaign vaccine is still available for those who have not received it yet but still want to. The vaccine contains inactivated, split influenza virus, propagated in eggs. One dose (0.5 ml) contains 3.75 μg antigen (haemagglutinin) from A/California/07/2009 (H1N1) strain. The AS03 adjuvant is composed of squalene (10.68 mg), DL-α-tocopherol (11.86 mg) and polysorbate 80 (4.86 mg). The vaccine contains 5 μg thiomersal.

In addition, the A/California/07/2009 (H1N1)-derived virus will be included in all trivalent seasonal influenza vaccines available in the country for the season 2010-11. The seasonal vaccine will be offered free of charge as part of the national vaccination program for adults at special risk because their health condition or medication and for persons more than 65 years of age. During the season 2010-11, new risk groups may be covered by the national program. The seasonal vaccines used in the national vaccination program 2010-11 will be Influvac® (Solvay) and Fluarix® (GlaxoSmithKline Biologicals). Influvac® contains surface antigens of influenza viruses produced in fertilised eggs and Fluarix® contains antigens of inactivated, split influenza viruses produced in eggs. One dose of both vaccines (0.5 ml) contains 15 μg antigen (haemagglutinin) of each of the three strains (H1N1, H3N2 and influenza B). In addition, there will be other comparable seasonal vaccines available in Finland by commercial products produced by several companies, and people may obtain these vaccines e.g. via private health care, occupational health care etc.

Thus, the most probable 2009 H1N1 vaccination regimens will be:

- one dose of the monovalent pandemic vaccination campaign vaccine (Pandemrix®) during the vaccination campaign October 2009 to May 2010 or at any time thereafter
- one dose of any of the seasonal vaccines during the season 2010-11
- one dose of the pandemic vaccination campaign vaccine Pandemrix during the vaccination campaign October 2009 to May 2010 and one dose of any of the seasonal vaccines during the season 2010-11
- no pandemic 2009 H1N1 vaccines

7 Enrolment

7.1 Enrollment in the interview follow-up phase of vaccinations and influenza like illness

Individuals participating in the original study AH1N1-483-09THL will be invited to participate in the current study by sending invitation letters home. The invitation letter will include the study information leaflet and the subjects are encouraged to ask questions by phone or email. In addition, the letter includes a short interview form, in which basic questions about previous 2009 H1N1 vaccinations, change in the baseline medical condition since the start of the original study AH1N1-483-09THL and potential pregnancy will be asked. The letter includes a consent form, and sending this signed form together with the filled interview form by mail, the subject enrolls in the interview follow-up phase of the study.

In the consent the study subjects give permission to report to the study staff, if they take either the monovalent 2009 H1N1 vaccine or a trivalent 2010-11seasonal influenza vaccine. They also consent to report to the study staff, if they have any symptoms of ILI as defined in chapter 5.1, as well as to make an appointment for laboratory confirmation of the 2009 H1N1 influenza cases. In addition, the consent includes permission to verify the vaccination data in medical records and to use the clinical and register data collected in the original study AH1N1-483-09THL in the analysis of the current study, if needed. The subjects are informed, that the collection of register data for assessing the effcetiveness of vaccination will be continued, according to the protocol and consent of the orginal study AH1N1-483-09THL.

7.2 Enrollment in the laboratory confirmation phase of 2009 H1N1 influenza cases

If a participant experiences symptoms and signs of ILI, a study visit will be arranged, at which a new informed, written consent for confirmation phase of 2009 H1N1 influenza cases will be obtained, including permission to take and analyse the study samples, collect details of the disease, verify the cases in medical records if needed, and combine the data collected during this phase with the data collected in the in the original study AH1N1-483-09THL.

8 Study outcome assessment

8.1 Assessment of the vaccination status in the interview follow-up

The participants are asked to report to the study staff, if they take either the monovalent 2009 H1N1 vaccine or the trivalent seasonal influenza vaccine 2010-11. They are contacted by SMS or phone monthly to ask about influenza vaccinations. For reporting and verification of vaccinations, a vaccination card will be sent to the subjects participating in the ínterview follow-up. The participant will be asked to request the vaccinator to fill in vaccination details in the card, if the subject gets any 2009 H1N1 influenza vaccine. If he/she fails to get the remarks from the vaccinator, the participant may fill the form himself/herself. In that case, the vaccination details are verified in medical records, if possible.

In addition, the vaccination data collected from the register of Tampere city in the original study AH1N1-483-09THL will be visited for purposes of the current study, if needed. All vaccinations included in the national vaccination program are provided or arranged by the municipal primary health care centre, which is obliged to record the vaccination details in the electronic health care data system.

8.2 Assessment of the occurrence of ILI in the interview follow-up

When an epidemic caused by the 2009 H1N1 pandemic influenza virus starts during the 2010-11 season in Finland, the participants in the ínterview follow-up will be sent a letter and asked to report to the study staff immediately, if they have any symptoms or signs of ILI defined in chapter 5.1. They are also contacted weekly by SMS or phone to ask about the occurrence of the symptoms. The follow-up is almost similar to the one the subjects have experienced in the study AH1N1-483-09THL. The subjects are sent a diary for recording details of the disease. They are also sent the information leaflet and a model of the consent form which will be signed at the study visit for the confirmation phase of 2009 H1N1 influenza cases.

8.3 Assessment of the laboratory-confirmed 2009 H1N1 influenza cases

For participants who report symptoms or signs of ILI defined in chapter 5.1 a study visit will be arranged, preferably within 3 days and at least within 7 days after the start of symptoms. At this visit, a written informed consent will be obtained for the confirmation phase for 2009 H1N1 influenza cases.

The acute phase visit will be arranged either at the home of the subject or at a special study clinic established for the study. Nasal and throat swabs will be obtained by a qualified study nurse or physician, to define the cases of laboratory confirmed 2009 H1N1 influenza (chapter 5.2). In addition, 10 ml of venous blood will be drawn at the acute phase. Clinical features of ILI will be recorded using the diary as an aid and a convalescent visit will be scheduled 2-3 weeks after the acute phase visit. Another diary will be given for follow up of the course of the disease. At the convalescent visit, 10 ml of venous blood will be drawn for measurement of the increase in 2009 H1N1 virus specific antibody levels, and further information of the disease will be collected using the diary as an aid.

8.3.1Assessment of the severity and possible complications of laboratory-confirmed 2009 H1N1 pandemic influenza cases

The clinical features of the disease and medical consultations will be asked at the acute and convalescence phase visits during ILI using the diaries as an aid.

To evaluate the severity of the disease, the data collected fom registers or medical records of Tampere city and TAUH may be visited for cases defined in chapter 5.2, if needed. If needed and possible, medical records will be requested from other than collaborating health care settings as well. Additional information on infections related to 2009 H1N1 influenza may be requested from the National Infectious Disease Register (e.g. invasive bacterial diseases).

8.3.2 Data from the registers of the health care centre, local hospitals and the National Infectious Disease Register

Data recorded in the electronic health care data systems during the visits of the study participants to Tampere health care centre, Tampere city hospital and Tampere University hospital (TAUH) and data from the National Infectious Disease Register of THL during the study period may be collected according to the protocol and consent of the original study AH1N1-483-09THL. These data comprise e.g. reasons of the visits, diagnoses of the patients, referrals to hospitals, policlinics and specialists, hospitalisations, emergency room visits and possible cases of death, laboratory records as well as individual medical records. The consent to use this data in the analysis of the current study will enable e.g. detection of potential missed cases from laboratory records and to analyse the visits temporally related to the cases verified in the study to explore the severity and possible complications.

9 Reporting of serious adverse events

No study spesific safety follow-up will be arranged because no vaccines are administered in the study, but the participants may or may not take vaccines as part of the normal medical practice. The safety is followed according to the national strategies for monitoring of the safety of 2009 H1N1 vaccination.

Finnish health care professionals nationwide are responsible for reporting serious and/or unexpected adverse reactions following immunisation (AEFI) to Unit of Vaccination Programme (ROHY) of the National Institute for Health and Welfare (THL) by using a form (AEFI form) designed specifically for this purpose (decree no. 421/2004 given by the Ministry of Social Affairs and Health). In addition, a specific form with pre-defined events with special interest has been established for reporting adverse events after vaccination with the 2009 H1N1 pandemic vaccine. The health care professionals send the AEFI report forms by mail or by fax to ROHY without undue delay. In urgent cases the reporting can be also done by phone. Additional or follow-up information relating to the initial suspected adverse event report is also to be completed and submitted with an AEFI form immediately, and medical records are requested, if needed. ROHY enters information on these reports to an electronic Register of Adverse Reactions Following Immunisation (AEFI register).

The vaccine safety professionals in ROHY analyse the AEFI cases for seriousness, unexpectedness and causality to vaccination with criteria compliant with ICH guidelines for Good Clinical Practice. All reports of serious adverse events after 2009 H1N1 vaccinations related to study participants will be delivered to the investigator without delay.

According to a national agreement, ROHY reports all serious adverse events (SAEs) reported to the AEFI register to the Finnish Medicines Agency. According to the national legislation, THL as the study sponsor will report to the Finnish Medicines Agency and the ethics committee of the Pirkanmaa Hospital District (PSHP) all SAE cases related to study participants for which the causal relationship with the 2009 H1N1 vaccine can not be ruled out and which are unexpected according to the Summary of Product Characteristics within time limits defined in the regulation of the Finnish Medicines Agency (LL Dnro 2740/0.5.1./2007). If the report has already been delivered to the Finnish Medicines Agency by ROHY, the documents will not be duplicated, but the report will only refer to the case and documents sent by ROHY. Once a year, THL will provide the Finnish Medicines Agency and the ethics committee of PSHP with a list of all SAE cases related to study participants for which the causal relationship with the 2009 H1N1 vaccine cannot be ruled out. The SAE reporting will last until the end of the follow-up.

10 Laboratory procedures

10.1 Laboratory methods for identifying of laboratory-confirmed 2009 H1N1 influenza cases

10.1.1 Nasal and throat swab samples

Viruses are intracellular pathogens. In order to obtain a good diagnostic sample for viral studies it must be attempted to collect as many epithelial cells as possible from the nostrils and the throat without causing excessive discomfort to the patient. To achieve maximal sensitivity, both nostrils and the throat must be swabbed. Nasal and throat

swab specimens will be obtained from individuals within 7 days after onset of ILI symptoms defined in chapter 5.1.

To obtain the nasal sample, a preferably flocked swab will be inserted approximately 3 – 4 cm into the first nostril, left in place for a few seconds, then slowly withdrawn with a rotating motion in order to trap as many epithelial cells as possible. If possible, the same swab will be used to sample also the other nostril. The swab will be broken into the tube containing transport medium. The throat, the tonsils, the uvula, and the posterior wall of the pharynx will be sampled using a fresh swab. This swab will be broken into the same tube containing the nasal swab. The sample will be placed in a refrigerator (temperature 4° C) for short-term storage, and/or in a freezer ($\leq$ -60° C) for extended storage until analysed. The samples will be sent to the laboratory by overnight mail in a small styrofoam box containing cool-cushions

In THL Virus Infection Unit (ROVI), 2009 H1N1 influenza viruses will be identified by reverse transcription polymerase chain reaction (RT-PCR) method and, if needed, by virus culture. For differential diagnostics and potential assessment for associations, influenza A and B as well as respiratory syncytial virus and potentially also other visuses causing respiratory infections will be identified from the combined nasal-throat swab specimens. Furthermore, influenza virus typing and subtyping may be performed using instructions of ROVI. Resistance to oseltamivir may be determined from selected samples.

RT-PCR methods for detecting influenza A (and B) virus-specific RNAs are readily available in the laboratory. Virus Infection Unit at THL functions as a National Influenza Center (NIC) and the unit carries out national surveillance work for the identification of circulating influenza virus strains in Finland. Approximately 1,000 nasopharyngeal specimens/year are screened and 150-200 influenza viruses identified and cultured each year. Influenza A virus-specific RT-PCR is based on general identification of influenza A viruses by matrix (M1) gene-specific primers that identify all influenza A viruses independent of their H or N gene subtype. Virus Infection Unit has also developed swine-origin pandemic influenza 2009 H1N1 specific RT-PCR assay based on H1 and NS1 gene specific primers that reliably enable the detection of the pandemic 2009 H1N1 virus infections. The assay was successfully used to determine the first influenza 2009 H1N1 infection cases in Finland in early May, 2009. Thus the

methodology for the rapid and specific influenza A virus diagnostics, for isolation and further characterization of influenza viruses are well established in the laboratory.

10.1.2 Blood samples for additional information on ILI cases

Of blood samples (á 10 ml) obtained at acute and convalescent phases of ILI, serum specimens will be prepared and stored at -20 °C or colder until analysed. The antibody titers will be measured using standard hemagglutination inhibition (ref 3) and neutralization tests (ref 4).

11 Statistical methods

11.1 Endpoints

The study endpoints for assessing the effectiveness of vaccination are defined in, respectively, 5.1 and 5.2. The primary endpoint will be laboratory-confirmed H1N1 influenza illness.

11.2 Sample size considerations

Out of 3500 persons in the original study, we anticipate to enrol 3000 persons to this second stage of the study. The estimated percentage of unvaccinated persons in this study cohort is approximately 50%, i.e. 1500. It is assumed that during the second wave of the 2009 H1N1 pandemic, which is the planned period of follow-up in this study, 5% of the unvaccinated become infected by the 2009 H1N1 virus, i.e. 75 expected cases. The assumed effectiveness of the vaccine is 70%. The study is powered to demonstrate the effectiveness of the vaccine such that the lower level of the 95% confidence interval is above 50%, which is regarded as the level of clinical relevance for the 2009 H1N1 vaccine. With a sample size of 3,000 the power of the study is 95%. High power for the effectiveness evaluation is preferred, since taking into account potential confounders, as well as possible unbalance in the age distribution of the study cohort (see section 3) will lower the power.

11.3 Statistical methods for analyses

The incidence of the laboratory-confirmed H1N1 pandemic influenza will be calculated as the number of cases divided by the person-time at risk, where person-time at risk is defined as compliance to the regular contacts. Periodic variation of incidence in calendar time will also be explored both in vaccinated and unvaccinated groups. Event-history analysis methods will be used for the evaluation of the effectiveness of the vaccine, where the risk of acquiring 2009 H1N1-influenza will be compared between the vaccinated/unvaccinated states, by accounting for calendar time and other potential confounders. Vaccine effectiveness will be calculated as 1- relative risk.. As an additional exploratory analysis, vaccine effectiveness will be also be estimated in subgroups stratified by age and sex. Due to the nature of the study (see section 3), other descriptive analyses and tests of hypotheses may also be carried out but the choice of subsequent hypotheses will be data dependent.

12 Data collection and management

12.1 Data entry

Clinical data collected at the study sites will be collected on paper CRFs or entered electronically during the visits. Software allowing direct validation of entry using defined entry criteria, crosschecks of various fields and control for missing data will be used. A paper back-up system will be available. The data may be optionally entered in an off-line mode, as the data entry may take place in various study sites. The electronic data will be incorporated into the study database daily using established systems for data transportation.

Bacteriologic and laboratory data will be entered manually after completion of the assays using the same software as for clinical data. Appropriate back-up files are maintained continuously.

12.2 Personal data and confidentiality

Each patient will maintain the same individual subject identification (ID) number as he/she had in the original study the original study AH1N1-483-09THL. Subjects' full identification information will be recorded in the database. The study related personal

and other confidential data will be managed confidentially, according to the national regulations and internal rules and guidelines of THL. Consents will be obtained to combine the data collected in the current study with that collected in the original study AH1N1-483-09THL.

12.3 Quality control

THL will perform internal quality control and monitoring. This will include education and training prior to study start, writing of study-specific SOPs and close follow-up of the study during the clinical phase. Study documentation and records retention will be performed according to the archiving rules of ICH and THL.

The compliance to the ICH guidelines for Good Clinical Practice and other quality requirements will be achieved by education of the study staff, according to the research tradition of the department, guidelines of the the handbook ' Good research practice in the National Public Health Institute' as well as other guidelines and internal regulations of the institute.

12.4 Discontinuation

If the subject withdraws his/her consent, the collection of data will be stopped. As main rule, the data recorded before the discontinuation of a subject will not be removed from the study database.

12.5 Archiving

The investigators' files and other study related documents are stored in locked cabinets in the study centre during the clinical phase, data resolution and analysis, as long as needed. Thereafter the documents are transposed to the final archive facilities of THL according to the institutional Archiving Guidelines and under supervision of the Senior Archivist of the institute. An archive index is maintained to record all research files that have been entered into the archive or destroyed. In general, all original research material generated at THL is retained permanently according to the national Archives Act (831/1994). THL will arrange access to the study documents during the follow up

and after archiving and collaborate with the relevant persons authorised to perform audits.

13 Institutions and investigators

Sponsor: National Institute for Health and Welfare (THL)

Principal investigators:

Principal investigator: T. Kilpi, M.D., Ph.D., Paediatrician, Head of Department of vaccination and immune protection, THL

Other investigators in Finland:

Jukka Jokinen, Ph.D., Statistician, THL

Tuija Leino, M.D., Ph.D., Senior researcher, THL

Ritva Syrjänen, M.D., Researcher, Clinical coordinator/study physician, THL

Jonas Sundman, Data management, THL

Laboratory analyses:

Virus Infection Unit, Department of Vaccination and Immune Protection

National Institute for Health and Welfare (THL), Helsinki, Finland

Ilkka Julkunen, M.D., Ph.D., Professor, Head

Thedi Ziegler, Ph.D., Laboratory Head

Jaana Pirhonen, Ph.D., Senior scientist

Mari Strengell, Ph.D., Research scientist

Esa Rönkkö, MSc., Research scientist

Niina Ikonen, MSc., Research scientist

Riitta Santanen, Lab. Technician

Anja Willberg, Lab. Technician

14 Ethical considerations

The study will be conducted according to the ICH guidelines for Good Clinical Practice (GCP), the Declaration of Helsinki, local rules and regulations of the country. The study protocol will be submitted to the ethics committee of the Pirkanmaa Hospital District (PSHP) and the Finnish Medicines Agency.

Although the aim of the study is to study 2009 H1N1 influenza vaccinations, the vaccines given during the study period are not an intervention introduced for the purposes of this study but part of the national vaccination strategy aiming at protecting the subjects from pandemic influenza. Thus no additional risk is created.

Written informed consent will be obtained from the subjects, separately for the interview follow-up by mail and for the confirmation of 2009 H1N1 influenza cases at the first sudy visit. The consents include the permission to verify details of the reported vaccinations and ILI cases from medical records and to use the data collected in the original study AH1N1-483-09THL for purposes of the current study, if needed.

The time needed for providing information by mail, phone and SMS during the interview follow-up is minor, about half an hour per month. To minimise the exertion caused by the study visit during the acute phase of ILI, the visit is offered to be performed at the home of the participant. Nasal and throat samples and blood samples to be collected in the study are generally accepted methods in clinical practice. The study procedures thus carry out minimal additional risk to the subjects as compared to normal clinical practices. The study procedures will be performed by experienced health care personnel only.

15 Time schedule of the study

The enrolment is planned to start in September 2010 and the follow-up of vaccinations soon thereafter, presumably in October 2010. The follow-up and laboratory confirmation of ILI cases will start immediately, when an epidemic of influenza caused by the 2009 H1N1 influenza virus or its variation is recognized to start in the region according to the influenza follow-up system of the Virus Infection Unit (ROVI) of THL provided the start of

the epidemic occurs by the end of March 2011. The follow-up will continue until the epidemic is considered to be over in the region, presumably by the end of April 2011. According to the protocol and consent of the original study AH1N1-483-09THL, the follow-up of register data can be extended. The laboratory analyses and statistical analyses are phased until the end of 2011. Uncertainty regarding the timing of epidemics necessitates the possibility for rapid re-evaluation of the study cohort enrollment plans and the length of follow-up, as is customary in these types of observational studies.

16 Publication of the results

Results of the study will be published as collaborative articles in scientific meetings and peer-reviewed medical journals with the principal investigator (TK) deciding on the authors and order, based on the proposals made by the research teams.

17 References

1. Lyytikäinen O., Kuusi M., Snellman M., Virtanen M.J., Rönkkö E., Ikonen N., Ziegler T., Julkunen I., Eskola J. and Ruutu M. Pandeeminen influenssa A (H1N1) 2009 - seurantatuloksia Suomesta. Suomen Lääkärilehti (Finnish Mecial Journal), in press, 2010.

2. Novel Swine-Origin Influenza A (H1N1) Virus Investigation Team. Emergence of a Novel Swine-Origin Influenza A (H1N1) Virus in Humans. N Engl J Med 2009 0: NEJMoa0903810

3. Ikonen N, Strengell M, Kinnunen L, Osterlund P, Pirhonen J, Broman M, Davidkin I, Ziegler T, Julkunen I. High frequency of cross-reacting antibodies against 2009 pandemic influenza A(H1N1) virus among the elderly in Finland. Euro Surveill. 2010 Feb 4;15(5). pii: 19478.

4. Lehtoranta L, Villberg A, Santanen R, Ziegler T. A novel, colorimetric neutralization assay for measuring antibodies to influenza viruses. J Virol Methods. 2009, 159:271-276.
